# Why does local edema persist? Immunological mismatch between phospholipase A_2_ potency and antivenom affinity in *Protobothrops mucrosquamatus* envenomation

**DOI:** 10.1371/journal.pntd.0014689

**Published:** 2026-09-03

**Authors:** Cheng-Hsuan Ho, Yu-Wei Chiang, Yan-Chiao Mao, Chih-Hsiung Hsu, Feng-Chen Chen, Shih-Hung Tsai, Shing-Hwa Liu

**Affiliations:** 1 Department of Emergency Medicine, Tri-Service General Hospital, National Defense Medical University, Taipei, Taiwan; 2 Institute of Toxicology, College of Medicine, National Taiwan University, Taipei, Taiwan; 3 Institute of Preventive Medicine, National Defense Medical University, New Taipei, Taiwan; 4 Department of Biology and Anatomy, National Defense Medical University, Taipei, Taiwan; 5 Department of Medical Toxicology, Taichung Veterans General Hospital, Taichung, Taiwan; 6 School of Public Health, National Defense Medical University, Taipei, Taiwan; 7 Department of Emergency Medicine, Kaohsiung Armed Forces General Hospital, Kaohsiung, Taiwan; 8 Department of Medical Research, China Medical University Hospital, China Medical University, Taichung, Taiwan; Fundação de Medicina Tropical Doutor Heitor Vieira Dourado: Fundacao de Medicina Tropical Doutor Heitor Vieira Dourado, BRAZIL

## Abstract

**Background:**

*Protobothrops mucrosquamatus* is the primary species responsible for snakebite morbidity in Taiwan, typically resulting in severe localized tissue edema. Although freeze-dried hemorrhagic antivenom (FHAV) is the standard treatment, persistent swelling remains a frequent clinical challenge. This study aimed to identify primary edematogenic toxins and evaluate the neutralizing efficacy of FHAV and small-molecule inhibitors (SMIs) to address this therapeutic gap.

**Methodology/principal findings:**

Multiple linear regression of a retrospective cohort of 50 patients was used to identify predictors of edema remission. Experimentally, a murine paw edema model was established to evaluate the potency of crude venom and isolated fractions (PLA_2_, SVSP, and SVMP/CTL), whereas ELISA was used to determine FHAV immunoreactivity. Clinically, total FHAV dosage was the strongest independent predictor of the remission timeline (*β* = 0.658, *T* = 5.167, *P* < 0.001), accounting for 69.8% of the variance. Experimentally, the PLA_2_-enriched fraction was identified as the primary edematogenic driver, eliciting swelling at 100 μg/mL, which was statistically indistinguishable from that of crude venom, whereas the other fractions required fivefold higher concentrations to achieve similar effects. However, ELISA revealed a significant immunological mismatch: the SVMP/CTL fraction sequestered 81.49% of the FHAV, indicating that highly potent PLA_2_ was poorly targeted. Furthermore, SMIs (varespladib, marimastat, and batimastat) at 10 μM exhibited limited efficacy against crude venom-induced edema, suggesting that single-target interventions against the complex venom proteome are insufficient.

**Conclusions/significance:**

Persistent localized edema arises from an immunological mismatch in which FHAV recognizes primarily high-molecular-weight immunogens instead of the primary edematogenic driver, PLA_2_. Current clinical management necessitates increasing FHAV dosages to compensate for low specific affinity through increased total antibody volume. These findings highlight that robust prospective multicenter trials are urgently needed to transition from empirical dosing to standardized, potency-aligned FHAV protocols, potentially incorporating multimodal therapeutic cocktails to achieve comprehensive neutralization.

## Author summary

Envenomation by *Protobothrops mucrosquamatus* is the leading cause of snakebite morbidity in Taiwan and frequently results in severe and persistent localized tissue edema. Although standard therapy with freeze-dried hemorrhagic antivenom (FHAV) is life-saving, clinicians regularly encounter a clinical paradox wherein patients experience progressive swelling despite receiving standard antivenom doses. This study elucidates this phenomenon by identifying a profound immunological mismatch between toxin potency and antivenom binding specificity. Mechanistically, phospholipase A_2_ (PLA_2_) enzymes serve as the primary drivers of rapid, intense edematogenesis, whereas FHAV targets predominantly higher molecular weight toxins with a lower relative capacity to induce tissue swelling. Retrospective analysis of a cohort of 50 patients and translational laboratory models confirmed that the total administered FHAV dose is the principal independent predictor of the edema remission timeline. These findings highlight that current antibody neutralization profiles are suboptimally aligned with the specific toxin families driving localized tissue destruction. Ultimately, this work advocates for a paradigm shift toward potency-aligned antivenom protocols and multimodal therapeutic cocktails to accelerate clinical recovery and minimize permanent localized tissue damage.

## Introduction

There are six medically important snake species in Taiwan, which are classified into two families, Viperidae and Elapidae. The family Viperidae includes *Protobothrops mucrosquamatus* (Günther, 1864), also known as the Taiwan habu, *Trimeresurus stejnegeri* (Schmidt, 1925), *Deinagkistrodon acutus* (Günther, 1888), and *Daboia siamensis* (Smith, 1917) [1,2]. The remaining two species, *Bungarus multicinctus* (Blyth, 1861) and *Naja atra* (Wüster, 1991), belong to the family Elapidae [1,2]. Among these species, *P. mucrosquamatus* is the species most frequently involved in snakebite incidents in Taiwan [3]. The clinical manifestations of *P. mucrosquamatus* envenomation are primarily severe localized tissue injury characterized by swelling and pain (96.2%), ecchymosis (55.5%), blistering or bullae (23.1%), wound infection (23.1%), and tissue necrosis (15.1%) [4]. These localized effects are significantly more prevalent than systemic symptoms are, which occur infrequently and are generally transient [4].

The proteome of *P. mucrosquamatus* venom is complex and includes four major protein families: snake venom metalloproteinases (SVMPs), ranging in content from 29.4–45.1%; phospholipase ${\text{A}}_{\text{2}}$ (PLA_2_), ranging from 15.9–25.0%; C-type lectin-like proteins (CTLs) or snake C-type lectins (Snaclecs), ranging from 12.8–21.1%; and snake venom serine proteases (SVSPs), ranging from 8.23–17.6%; as well as other components, such as hyaluronidase and L-amino acid oxidase (LAAO) [4–7]. SVMPs play a critical role in the destruction of muscle cells and tissue, primarily manifesting as myonecrosis and permanent functional impairment [8]. While muscle damage has historically been attributed mainly to PLA_2_, recent evidence shows that P-I class SVMPs are closely involved in the persistent disruption of skeletal muscle fibers [9,10]. The mechanisms of destruction are both direct and indirect; SVMPs directly degrade the extracellular matrix (ECM) and basement membrane components that maintain the structural integrity of muscle fibers while also inducing secondary myonecrosis through ischemia and hypoxia caused by the rapid rupture of surrounding microvessels, resulting in hemorrhage [11]. Furthermore, SVMPs impede skeletal muscle regeneration by disrupting the local microenvironment and affecting muscle stem cell precursors, often leading to the replacement of muscle tissue with permanent fibrosis [11]. Additionally, SVMPs trigger the release of endogenous inflammatory mediators, including IL-1β, IL-6, and matrix metalloproteinases (MMPs), which exacerbate vascular permeability [12]. PLA_2_ subtypes are categorized on the basis of their primary structural subtype, specifically the catalytically active Asp49 (D49) variants and the catalytically inactive but pharmacologically potent homologs, including the Lys49 (K49), Asn49 (N49), and novel Arg49 (R49) subtypes [13–15]. Functionally, these PLA_2_ isoforms exhibit broad-spectrum toxicity: basic D49 variants such as trimucrotoxin induce potent presynaptic neurotoxicity by blocking acetylcholine release [13,16]; the noncatalytic K49 and R49 subtypes drive localized myonecrosis and intense edema through direct membrane disruption and the promotion of mast cell degranulation [4,15,17]. CTL or Snaclecs, which induce platelet aggregation, include mucetin [18], mucrocetin [18], trimecetin [6], and trimucytin [4]. Snake venom serine proteinases (SVSPs) exhibit two primary functions: first, they degrade fibrinogen, thereby inducing coagulopathy [6]; and second, certain SVSPs release bradykinin, which causes vasodilation and increases capillary permeability, collectively culminating in severe tissue edema [4]. Furthermore, other components, such as hyaluronidase and L-amino acid oxidase (LAAO), exert synergistic effects with PLA_2_ and SVMPs in the pathogenesis of edema [19]. Although progressive swelling is the hallmark clinical presentation of *P. mucrosquamatus* envenomation, the specific toxins that primarily play an edematogenic role and the precise nature of their synergistic interactions remain unknown.

In Taiwan, freeze-dried hemorrhagic antivenom (FHAV) is the standard treatment for *P. mucrosquamatus* and *T. stejnegeri* bites [20]. FHAV is a lyophilized, bivalent, equine-derived F(ab’)_2_ fragment antivenom [21]. Adverse reactions following administration remain relatively uncommon [22]. Early complications occurring within 1 h of infusion primarily include mild allergic responses such as rash, pruritus, or pyrogenic reactions, with an overall incidence of approximately 3% even among patients with positive skin test results [20,22]. Delayed complications, predominantly serum sickness, typically manifest 4–14 d after administration, with the risk increasing markedly at cumulative FHAV doses exceeding 20 vials [22]. Although serum venom antigen levels decrease significantly within 6 h of FHAV administration and become undetectable in approximately 57.1% of patients [23], definitive clinical guidelines determining the necessity for supplemental FHAV administration are currently lacking. Clinicians often rely on empirical approaches, such as sonographic assessment of the rate of proximal progression or gross visual evaluation [23,24]. However, clinical practice has revealed a paradox wherein many patients continue to experience progressive swelling despite receiving FHAV [24]. This uncertainty underscores whether FHAV effectively neutralizes *P. mucrosquamatus*-induced localized tissue injury. A similar therapeutic gap has been documented for *Naja atra* bites, where freeze-dried neurotoxic antivenom (FNAV; Taiwan Centers for Disease Control, Taipei, Taiwan) was found to be ineffective at preventing venom-induced dermonecrosis in animal models [25,26]. This study aims to evaluate the neutralizing efficacy of FHAV against *P. mucrosquamatus*-induced edema and test the hypothesis that persistent swelling arises from the synergistic effects of toxins that are not uniformly neutralized by current therapy.

## Materials and methods

### Ethics statements

**Institutional Review Board (IRB):** The study protocol, informed consent forms, and participant information sheets were reviewed and approved by the Institutional Review Board of Tri-Service General Hospital (IRB No. 2-107-05-039). The principal investigator, *Cheng-Hsuan Ho*, was responsible for reporting all serious adverse events (SAEs) and unanticipated problems in compliance with applicable regulatory requirements. The IRB operates in strict accordance with Good Clinical Practice (GCP) guidelines and relevant national laws.

**Informed Consent:** Written informed consent for the collection and publication of clinical data was obtained from all participants or their legally authorized representatives prior to inclusion in this study.

**Animal Ethics (IACUC):** All animal experimental procedures were reviewed and approved by the Institutional Animal Care and Use Committee (IACUC) of the National Defense Medical University, Taiwan (Protocol No. IACUC-24–130).

### Clinical patients

Snakebite patients who were admitted to the emergency department of Tri-Service General Hospital from 2017 to 2025 were included in this study, following approval obtained from the Institutional Review Board of Tri-Service General Hospital (IRB No. 2-107-05-039). Of the 81 patients enrolled, 50 were identified as individuals envenomated by *P. mucrosquamatus* on the basis of the clinical identification of the snake carcass or digital photographs provided by the patient. This study employed a retrospective analytical design to evaluate the clinical predictors of recovery in these 50 patients envenomated by *P. mucrosquamatus*. To identify factors influencing the duration of local edema remission, a multiple linear regression model incorporating 12 independent clinical variables was constructed using the Enter method. Demographic variables included sex and age, whereas pre-existing comorbidities were defined as the presence of diabetes mellitus or chronic kidney disease (CKD) stages 1–5 according to the KDIGO 2026 Clinical Practice Guideline [27]. Clinical presentation was characterized by the bite-to-emergency department (ED) time in hours and the anatomical bite site. Edema severity was quantified using the Blaylock classification at two critical time points: the initial Blaylock level upon arrival and the final Blaylock level, which represents the peak of swelling progression, ranging from minimal to gross edema [28]. The Blaylock classification defines clinical severity as minimal for localized edema, mild for swelling restricted to the hand or foot, moderate for edema extending from the hand to the shoulder or from the foot to the thigh, severe for progression from the hand to the chest or from the foot to the groin, and gross for swelling extending from the foot to the trunk or from the hand to the contralateral chest [28]. Treatment and hospitalization factors were defined as the total dosage of FHAV administered, the duration of ED stay in hours, the need for surgical intervention, and the duration of hospital admission in days. The dependent variable for this predictive model was remission duration, defined as the total time in days required for the complete resolution of venom-induced localized edema, which was documented through patient recall during telephone follow-up interviews or routine outpatient clinical visits.

### Reconstitution and chromatographic fractionation of venom reagents

Lyophilized crude *P. mucrosquamatus* venom was provided by the Department of Biology and Anatomy at National Defense Medical University (Taipei, Taiwan). Upon receipt, the lyophilized venom was divided into single-use aliquots and stored at −20°C until use. This procedure was adopted to standardize sample handling and minimize potential variability associated with prolonged storage and repeated freeze‒thaw cycles [29,30]. Immediately before each experiment, an individual aliquot was reconstituted in sterile phosphate-buffered saline (PBS; Gibco [Thermo Fisher Scientific, Waltham, MA, USA]) to the required concentration. To remove particulate matter, the solution was gently vortexed for 10 s and centrifuged at 10,000 × *g* for 5 min at 4°C using an Eppendorf 5424 R centrifuge (Eppendorf AG, Hamburg, Germany). The clarified supernatant was filtered through a 0.22 μm PVDF syringe filter (Millipore, Burlington, MA, USA) and divided into single-use aliquots, ensuring that each venom aliquot was subjected to only one use to avoid freeze-thaw degradation or loss of enzymatic activity. The protein concentration was quantified using a bicinchoninic acid (BCA) protein assay kit (Thermo Fisher Scientific, Waltham, MA, USA) with bovine serum albumin (BSA; Sigma‒Aldrich, St. Louis, MO, USA) as a standard, and calibration curves were constructed using serial dilutions of BSA ranging from 0–2,000 µg/mL. Unless otherwise specified, all the reagents used were of analytical or HPLC grade, and all the injectable and chromatographic solutions were prepared using ultrapure water generated from a Direct-Q water purification system (Millipore, Burlington, MA, USA) and passed through 0.22 µm PVDF syringe filters prior to use. For subsequent protein isolation, the clarified crude *P. mucrosquamatus* venom was fractionated on the basis of molecular mass using a Superdex 200 Increase 10/300 GL column connected to an ÄKTA Pure chromatography system (Cytiva, Uppsala, Sweden). The column was equilibrated and eluted with 50 mM phosphate buffer containing 150 mM NaCl (pH 7.4) at a flow rate of 0.5 mL/min, and the elution profile was monitored at 280 nm. Fractions corresponding to the same peaks were pooled and tentatively assigned to the peptide-enriched fraction (< 10 kDa), the PLA_2_-enriched fraction (approximately 14 kDa), the SVSP-enriched fraction (approximately 30 kDa), and a high-molecular-weight SVMP/CTL-enriched fraction (P-II or P-III; > 50 kDa). Because snake C-type lectin (Snaclec/CTL) is often present in a multimeric form with a molecular weight of approximately 121 kDa, it was not specifically isolated in this experiment but was instead cofractionated with snake venom metalloproteinase, and this fraction was labeled SVMP/CTL. Each pooled fraction was then lyophilized using a FreeZone 2.5 L freeze dryer (Labconco, Kansas City, MO, USA) and stored at −20°C with desiccant until further use. To confirm the composition and hydrophobicity profiles, each size-exclusion chromatography (SEC)-derived fraction was reconstituted in 0.1% trifluoroacetic acid (TFA) and analyzed using an Agilent 1260 Infinity II HPLC system (Agilent Technologies, Santa Clara, CA, USA) equipped with a Jupiter C18 column (250 × 4.6 mm, 5 μm particle size, 300 Å pore size; Phenomenex, Torrance, CA, USA). Mobile phase A consisted of water containing 0.1% TFA, and mobile phase B consisted of HPLC-grade acetonitrile containing 0.1% TFA. The proteins were eluted using a linear gradient from 5–70% mobile phase B over 85 min at a flow rate of 1.0 mL/min with monitoring at 215 nm. The fraction assignments in this study were established on the basis of a previous study [6] in which the major snake venom protein families, including PLA_2_, SVSP, SVMP, and CTL, were thoroughly mapped using liquid chromatography-tandem mass spectrometry (LC-MS/MS) and characterized under identical chromatographic conditions. Although referencing these established profiles provided a robust baseline for defining elution windows, the specific pooled fractions utilized in the present experiments were not independently reconfirmed by LC-MS/MS, SDS-PAGE, or toxin-family-specific enzymatic assays.

### Oversight, ethical approval for the animal study and animal housing

All procedures involving animals were reviewed and approved by the Institutional Animal Care and Use Committee (IACUC) of the National Defense Medical University, Taiwan (protocol number IACUC-24–130) and were conducted in strict accordance with institutional guidelines and national regulations governing the ethical use of laboratory animals. Every effort was made to minimize animal suffering and reduce the total number of animals used in the study. Because acute inflammatory edema served as the primary experimental endpoint, no preemptive analgesics were administered prior to paw thickness measurements to prevent pharmacological interference with the release of inflammatory mediators or changes in vascular permeability. Male ICR/CD-1 mice between 6–8 weeks of age weighing 20 ± 2 g obtained from BioLASCO Taiwan Co., Ltd. (Taipei, Taiwan) were utilized for all experiments. These animals were housed under standard facility conditions, including a 12-h light/dark cycle at an ambient temperature of 22 ± 2°C and a relative humidity between 40% and 60%. The mice were acclimatized for at least 7 d with *ad libitum* access to standard laboratory chow and water and housed in groups of 3–5 per cage with corncob bedding. Before venom or its isolated fractions was administered, the mice were briefly anesthetized with 2%–3% isoflurane (Forane; Baxter Healthcare Corporation, Deerfield, IL, USA) delivered in oxygen to minimize handling stress. All the animals recovered immediately following the subplantar injection procedure and were monitored closely for any signs of abnormal behavior or local tissue damage. This intensive postinjection monitoring ensured rapid recovery and the total absence of distress in strict compliance with Institutional Animal Care and Use Committee regulations, and humane endpoints were predefined and monitored throughout the entire experimental period.

### Establishment of the paw edema model and characterization of venom- and fraction-induced local edema

The local inflammatory response to *P. mucrosquamatus* venom and its isolated components was evaluated using a modified subplantar paw edema assay in mice (*n* = 6 per group). These group sizes were determined on the basis of the initial variability in the paw thickness measurements, which was deemed sufficient to characterize dose-dependent and treatment-related effects while strictly minimizing animal usage in accordance with ethical guidelines, although a formal *a priori* power calculation was not performed. Baseline paw thickness was measured at the central plantar pad using digital calipers (Mitutoyo Digimatic [0.01 mm resolution]; Mitutoyo Corp., Kawasaki, Japan) prior to treatment. Under brief isoflurane anesthesia, the animals received a 50 µL subplantar injection of either crude venom (CV) at various concentrations (10, 50, 100, 250, 500, or 1,000 µg/mL, equivalent to 0.5–50 µg per paw) or protein mass-matched doses of isolated toxin fractions. These fractions included PLA_2_ at 50 or 100 µg/mL, SVSP at 250 or 500 µg/mL, and SVMP/CTL at 250 or 500 µg/mL, with saline serving as the vehicle control. Paw thickness was recorded 3 h postinjection at the same anatomical location, a time point corresponding to maximal swelling as determined by preliminary kinetic observations. All measurements were performed in triplicate for each animal to calculate a mean value for analysis. The experimental results are expressed as the mean ± SD.

### Neutralization of venom-induced edema by antivenom and small-molecule inhibitors *in vivo*

The neutralizing efficacy of freeze-dried hemorrhagic antivenom (FHAV; Taiwan Centers for Disease Control, Taipei, Taiwan; lot number 60-06-0020) and small-molecule inhibitors against venom-induced edema was evaluated in a murine model. To minimize potential batch-to-batch variability, all the experiments were conducted using a single lot of antivenom, which was stored at 4°C and prepared immediately before use according to the manufacturer’s instructions. Mixtures containing CV and FHAV were preincubated at 37°C for 30 min with gentle agitation at 300 rpm using a ThermoMixer C (Eppendorf AG, Hamburg, Germany) prior to administration. Each 50 µL mixture was administered via subplantar injection into the left hind paw of mice under brief isoflurane anesthesia (*n* = 6 per group). The control groups received either antivenom alone or CV at a concentration of 100 µg/mL. The small-molecule inhibitors (SMI) utilized in this study included marimastat (Cat. No. SI-M2699; Selleck Chemicals, Houston, TX, USA), batimastat (Cat. No. S7036; Sigma‒Aldrich, St. Louis, MO, USA), and varespladib (Cat. No. HY-13402; MedChemExpress, Monmouth Junction, NJ, USA). All stock solutions were prepared according to the manufacturers’ instructions and diluted to the target concentrations immediately before the experiment. For the inhibitor assays, the three SMIs were prepared at a concentration of 10 µM, mixed with 100 or 250 µg/mL CV, and preincubated under the same temperature and agitation conditions as the pure antivenom (*n* = 3 per group). Paw thickness was recorded at baseline and 3 h postinjection using digital calipers (Mitutoyo Corp., Kawasaki, Japan).

### Characterization of the antivenom binding profiles via an indirect enzyme-linked immunosorbent assay (ELISA)

Venom samples, including CV and purified fractions such as PLA_2_, SVSP, and SVMP/CTL, were diluted in carbonate–bicarbonate buffer (0.05 M, pH 9.6) to a final concentration of 10 µg/mL. Each well of a flat-bottom 96-well ELISA plate (Nunc MaxiSorp; Thermo Fisher Scientific, Waltham, MA, USA) was coated with 100 µL of the antigens and incubated overnight at 4°C. After the wells were washed with phosphate-buffered saline containing 0.05% Tween-20 (PBST), the wells were blocked with 5% bovine serum albumin (BSA) in PBS for 1 h at room temperature. Reconstituted FHAV was serially diluted 1:500, 1:5,000, and 1:50,000 in blocking buffer, and 100 µL was added to the wells in triplicate, followed by incubation at 37°C for 1 h. After the wells were washed, bound equine antibodies were detected using horseradish peroxidase (HRP)-conjugated goat anti-horse IgG (Jackson ImmunoResearch Laboratories, West Grove, PA, USA) diluted 1:10,000 for 1 h at 37°C. The wells were subsequently washed, and the signal was developed using 3,3,’5,5’-tetramethylbenzidine (TMB) substrate (Thermo Fisher Scientific, Waltham, MA, USA) for 10 min in the dark. The reaction was terminated by the addition of 1 N H_2_SO_4_ (Sigma‒Aldrich, St. Louis, MO, USA), and the absorbance was measured at 450 nm using a Synergy H1 microplate reader (BioTek Instruments, Winooski, VT, USA). Background wells without FHAV were included for normalization, and the results are presented as background-subtracted optical density (OD) values at 450 nm.

### Statistical methods

Statistical analyses were performed using IBM SPSS Statistics version 23 (IBM Corp., Armonk, NY, USA) and GraphPad Prism version 11.0.1 (GraphPad Software, San Diego, CA, USA). Descriptive statistical data are summarized as the mean ± standard deviation (SD) or frequency for all the variables. Clinical predictors were evaluated using Pearson’s correlation and multiple linear regression models. A mixed-effects model was employed to analyze the edematogenic responses to CV and isolated toxin families, including PLA_2_, SVSP, and SVMP/CTLs, as well as the results of neutralization assays involving FHAV or SMIs. The experimental design utilized *n* = 6 mice per group for CV titration and FHAV neutralization, whereas *n* = 3 mice per group were used for SMI testing. Although cohort sizes were selected pragmatically without formal *a priori* power calculations, the group size of six mice balanced the ethical reduction in animal usage with the statistical reliability required to accommodate observed interanimal biological variability in paw thickness measurements, providing consistent and statistically detectable responses. To compare ELISA results across multiple dilution factors, two-way ANOVA was performed. Tukey’s post hoc test was used for multiple comparisons to analyze significant differences between treatment groups, time points, and antigens. All ELISA experiments were conducted in triplicate, with saline or designated negative controls serving as references. For all analyses, statistical significance was defined as *P* < 0.05.

## Results

### Clinical predictors and multivariate analysis of the duration of remission in patients with *Protobothrops mucrosquamatus* envenomation

The descriptive statistics for the study cohort of 50 patients envenomated by *P. mucrosquamatus* who presented to the ED of Tri-Service General Hospital from 2017 to 2025 are summarized in Table 1. The study population (*n* = 50) had a mean age of 53.24 ± 19.6 years (range: 5–88) and was predominantly male (*n* = 34, 68%). Comorbidities were present in a minority of the patients, including diabetes mellitus (*n* = 2, 8%) and chronic kidney disease (*n* = 28, 56% for stage 1; *n* = 13, 26% for stage 2; *n* = 9, 18% for stage 3). The mean time from bite to ED arrival was 3.17 ± 5.47 h (range: 0–29 h, with 0 hours representing an arrival within 30 minutes of envenomation), with the foot being the most frequent bite site (*n* = 23, 46%). The initial Blaylock severity scores were categorized as minimal in 40% of the patients, mild in 48%, and moderate in 12%, which progressed to peak severity categorized as minimal in 12% of the patients, mild in 54%, moderate in 32%, and severe in 2% during the clinical course. Treatment involved a mean FHAV dosage of 6.52 ± 4.24 vials (range: 1–22) and a mean ED stay of 19.69 ± 11.63 h, with the mean duration for complete local edema remission being 10.80 ± 10.88 d. Univariate analysis using Pearson’s correlation identified total FHAV dosage (*r* = 0.743, *P* < 0.001), ED stay duration (*r* = 0.544, *P* < 0.001), and final Blaylock level (*r* = 0.375, *P* = 0.004) as factors significantly associated with recovery time. To identify independent predictors, a multiple linear regression analysis was conducted, yielding a highly significant model (F_12, 37_ = 7.128, *P* < 0.001) with an *R*^*2*^ of 0.698. Within this model, total FHAV dosage emerged as the strongest independent predictor of the remission timeline (*β* = 0.658, *T* = 5.167, *P* < 0.001), followed by hospital admission duration (*β* = 0.355, T = 3.274, *P* = 0.002) and initial Blaylock level (*β* = - 0.283, *T* = - 2.039, *P* = 0.049), whereas age (*P* = 0.434), bite-to-ED time (*P* = 0.316), and comorbid diabetes mellitus (*P* = 0.067) were not independent predictors. Diagnostic tests confirmed the stability of the model, with all variance inflation factors remaining below 2.4. Taken together, these findings demonstrate that the total FHAV dosage serves as the primary clinical indicator of the duration of venom-induced localized edema.

**Table 1:** Demographic and clinical characteristics of 50 patients envenomated by *Protobothrops mucrosquamatus.*

Characteristic	n (%)	Mean	SD
**Gender**			
Male	34 (68.0)	–	–
Female	16 (32.0)	–	–
**Age** (years)	–	53.24	19.60
**Diabetes mellitus**	4 (8.0)	–	–
**Chronic kidney disease stage** ^1^			
Stage 1	28 (56.0)	–	–
Stage 2	13 (26.0)	–	–
Stage 3	9 (18.0)	–	–
**Bite-to-ED time** (h)	–	3.17	5.47
**Bite site**			
Finger	5 (10.0)	–	–
Hand	11 (22.0)	–	–
Forearm	1 (2.0)	–	–
Thigh	1 (2.0)	–	–
Lower leg	4 (8.0)	–	–
Foot	23 (46.0)	–	–
Toe	5 (10.0)	–	–
**Initial Blaylock level** ^2^			
Minimal	20 (40.0)	–	–
Mild	24 (48.0)	–	–
Moderate	6 (12.0)	–	–
**Final Blaylock level** ^**2**^			
Minimal	6 (12.0)	–	–
Mild	27 (54.0)	–	–
Moderate	16 (32.0)	–	–
Severe	1 (2.0)	–	–
**FHAV dosage** (vials)	–	6.52	4.24
**ED stay** (h)	–	19.69	11.63
**Surgical intervention**	1 (2.0)	–	–
**Hospital admission** (n = 7, days)	–	10.28	5.39
**Edema remission** (days)	–	10.80	10.88

^1^: Chronic kidney disease stage was classified based on the estimated glomerular filtration rate (eGFR) criteria.

^2^: Blaylock classification of edema severity: minimal, localized edema; mild, swelling confined to the hand or foot; moderate, edema extending from the hand to the shoulder or from the foot to the thigh; severe, progression from the hand to the chest or from the foot to the groin; gross, swelling extending from the foot to the trunk or from the hand to the contralateral chest.

Abbreviations: ED, emergency department; FHAV, freeze-dried hemorrhagic antivenom; SD, standard deviation.

### Characterization and validation of the dose-dependent edematogenic potential of *Protobothrops mucrosquamatus* crude venom in a mouse model

**Fig 1:**
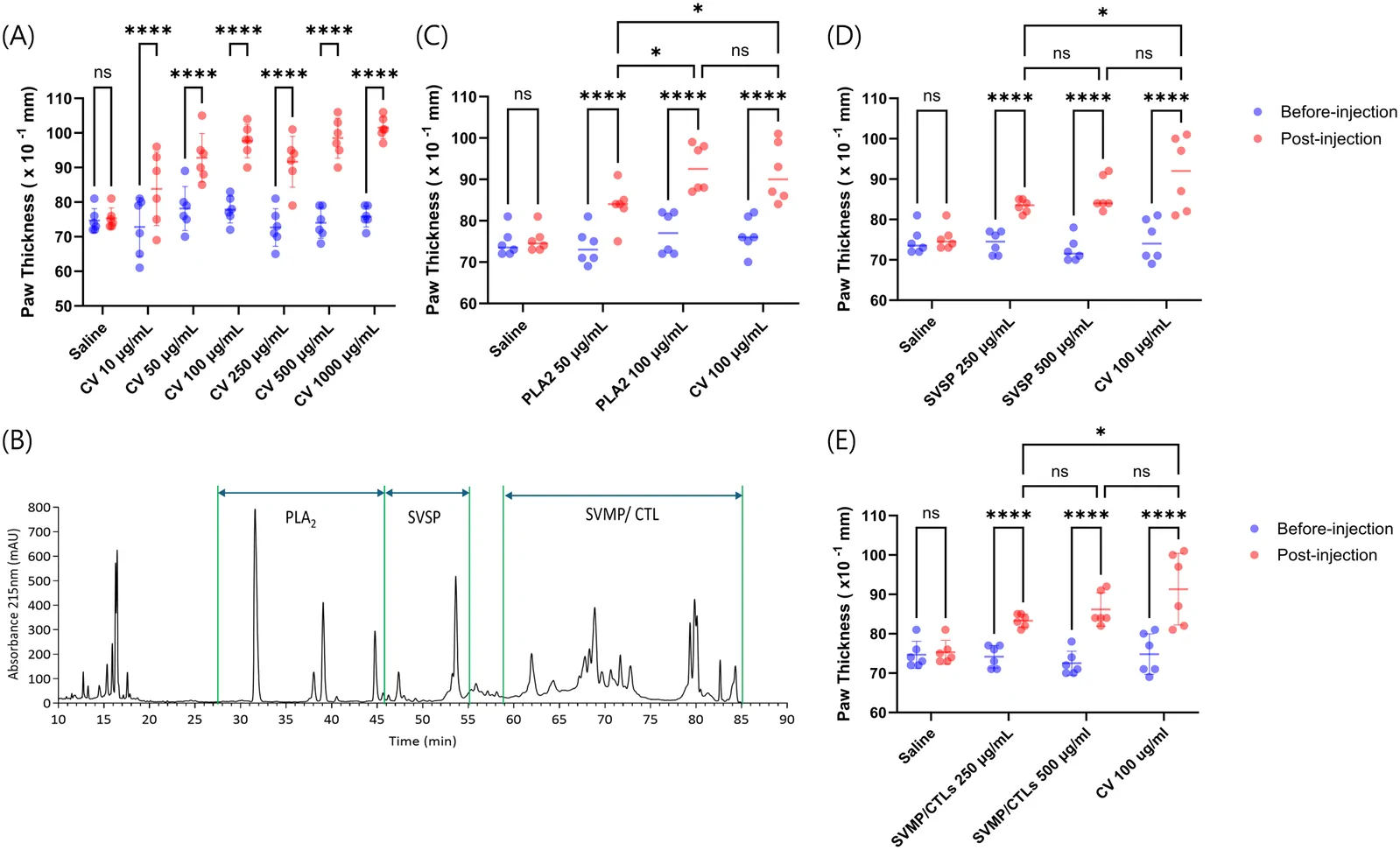
Dose-dependent edematogenic profile and chromatographic toxin fractionation of *Protobothrops mucrosquamatus* crude venom (CV). **(A)** Representative gross morphological changes and quantitative evaluation of the murine paw edema models following subplantar injection of crude venom (CV; 10–1,000 μg/mL), demonstrating rapid, concentration-dependent localized swelling at 3 h postinjection compared with saline controls. **(B)** Chromatographic fractionation of CV using reversed-phase high-performance liquid chromatography (RP-HPLC), with the proteome resolved into three primary toxin-enriched fractions on the basis of elution time: phospholipase A_2_ (PLA_2_-enriched; 27.5–46 min), snake venom serine protease (SVSP-enriched; 46–55 min), and snake venom metalloproteinase with C-type lectin-like proteins (SVMP/CTL-enriched; 59–85 min). **(C)** Edematogenic activity of the PLA_2_-enriched fraction, which was identified as the main driver of edema because a low dose (100 μg/mL) elicited a response statistically indistinguishable from that of 100 μg/mL CV (*P* = 0.9767). **(D and E)** Edematogenic characterization of the SVSP-enriched and SVMP/CTL-enriched fractions, respectively, showing that while both elicit significant edema (*P* < 0.0001), they are both less potent than the PLA_2_-enriched fraction is and require a fivefold higher concentration (500 μg/mL) to match the swelling magnitude of either the PLA_2_ fraction or CV (*P* = 0.2236). Baseline measurements are indicated by blue circles, and 3 h postinjection values are indicated by red circles, with quantitative data presented as individual points representing the mean ± standard deviation (SD). All the statistical comparisons were performed using a mixed-effects model followed by Tukey’s *post hoc* test (*n* = 6 per group; * *P* < 0.05, ** *P* < 0.01, *** *P* < 0.001, **** *P* < 0.0001 versus baseline).

Based on clinical findings, a murine paw edema model was established to simulate the progressive localized swelling observed in envenomated patients. Animals received subplantar injections of graded concentrations of CV into the hind paw, and paw thickness was subsequently recorded at 3 h post-injection at the identical anatomical location (Fig 1A). A mixed-effects model analysis revealed a highly significant interaction between intervention and time (*F*_6, 35_ = 137.7, *P* < 0.0001) and a predominant main effect of time (*F*_1, 35_ = 3,490, *P* < 0.0001). While the saline control group exhibited no significant change in paw thickness from baseline (7.467 mm to 7.533 mm; *q* = 1.278, *P* = 0.3724), subplantar injection of CV at all tested concentrations (ranging from 10 μg/mL to 1,000 μg/mL) induced profound and statistically significant localized edema. The magnitude of swelling was strictly dose dependent; specifically, the mean paw thickness of the 10 μg/mL CV group increased by 1.000 mm (*q* = 21.08, *P* < 0.0001), whereas that of the 1,000 μg/mL CV group exhibited a substantially greater increase of 2.567 mm (*q* = 49.19, *P* < 0.0001). At 3 h postinjection, concentrations of 50 μg/mL or more resulted in a paw thickness that was significantly greater than that in the saline control group (saline vs. 50 μg/mL CV, *q* = 7.242, *P* < 0.0001; saline vs. 1,000 μg/mL CV, *q* = 10.83, *P* < 0.0001). These findings demonstrate that *P. mucrosquamatus* CV triggers rapid, high-magnitude localized tissue swelling that scales precisely with venom concentration, thereby providing a robust and sensitive model for evaluating the edematogenic potency of specific toxin families.

### Comparative edematogenic potency of isolated PLA_2_-enriched, SVSP-enriched, and SVMP/CTL-enriched fractions from *Protobothrops mucrosquamatus* venom

To characterize the venom constituents, reversed-phase high-performance liquid chromatography (RP-HPLC) was performed to resolve the major toxin families of *P. mucrosquamatus* venom, yielding PLA_2_-enriched, SVSP-enriched, and SVMP/CTL-enriched fractions on the basis of established chromatographic profiles [6]. As illustrated in the chromatographic profile (Fig 1B), PLA_2_-enriched variants eluted in the first major region (approximately 27.5 min to 46 min), followed by the SVSP-enriched fraction from 46 min to 55 min. The most hydrophobic components, identified as the SVMP/CTL-enriched fraction, eluted in the final phase from 59 min to 85 min. The localized edematogenic activities of these three major toxin fractions were evaluated using a mouse paw edema model (Fig 1C–1E). For the PLA_2_-enriched fraction (Fig 1C), a mixed-effects model revealed a highly significant interaction between treatment and time (*F*_3, 20_ = 43.18, *P* < 0.0001). Post hoc analysis indicated that both the 50 μg/mL and 100 μg/mL doses induced marked swelling relative to baseline, with mean differences in volume of 0.983 mm and 1.583 mm, respectively (*P* < 0.0001 for both). Similarly, analyses of the SVSP-enriched and SVMP/CTL-enriched fractions revealed a significant interaction between treatment and time (F_3, 20_ = 37.52, *P* < 0.0001 for both), with doses of 250 μg/mL and 500 μg/mL resulting in substantial increases in paw thickness (*P* < 0.0001 for all). Collectively, these results indicate that while all three toxin fractions strongly induce localized edema, their relative potencies differ significantly. The PLA_2_-enriched fraction emerged as the most potent component; a dose of only 100 μg/mL elicited an edematous response (mean difference: 1.583 mm) that was statistically indistinguishable from that of the 100 μg/mL CV control (*P* = 0.9767). In contrast, 5-fold greater concentrations of the SVMP/CTL-enriched and SVSP-enriched fractions (500 μg/mL) were needed to produce comparable swelling (mean difference: 1.367 mm) and were statistically similar to the CV control (*P* = 0.2236 for both). These findings suggest that multiple toxin classes contribute to the local tissue damage characteristic of *P. mucrosquamatus* envenomation, with the PLA_2_ family serving as the primary driver of rapid, high-magnitude edema at relatively low protein concentrations.

### Neutralizing efficacy of freeze-dried hemorrhagic antivenom against *Protobothrops mucrosquamatus* venom-induced edema in a murine model

**Fig 2:**
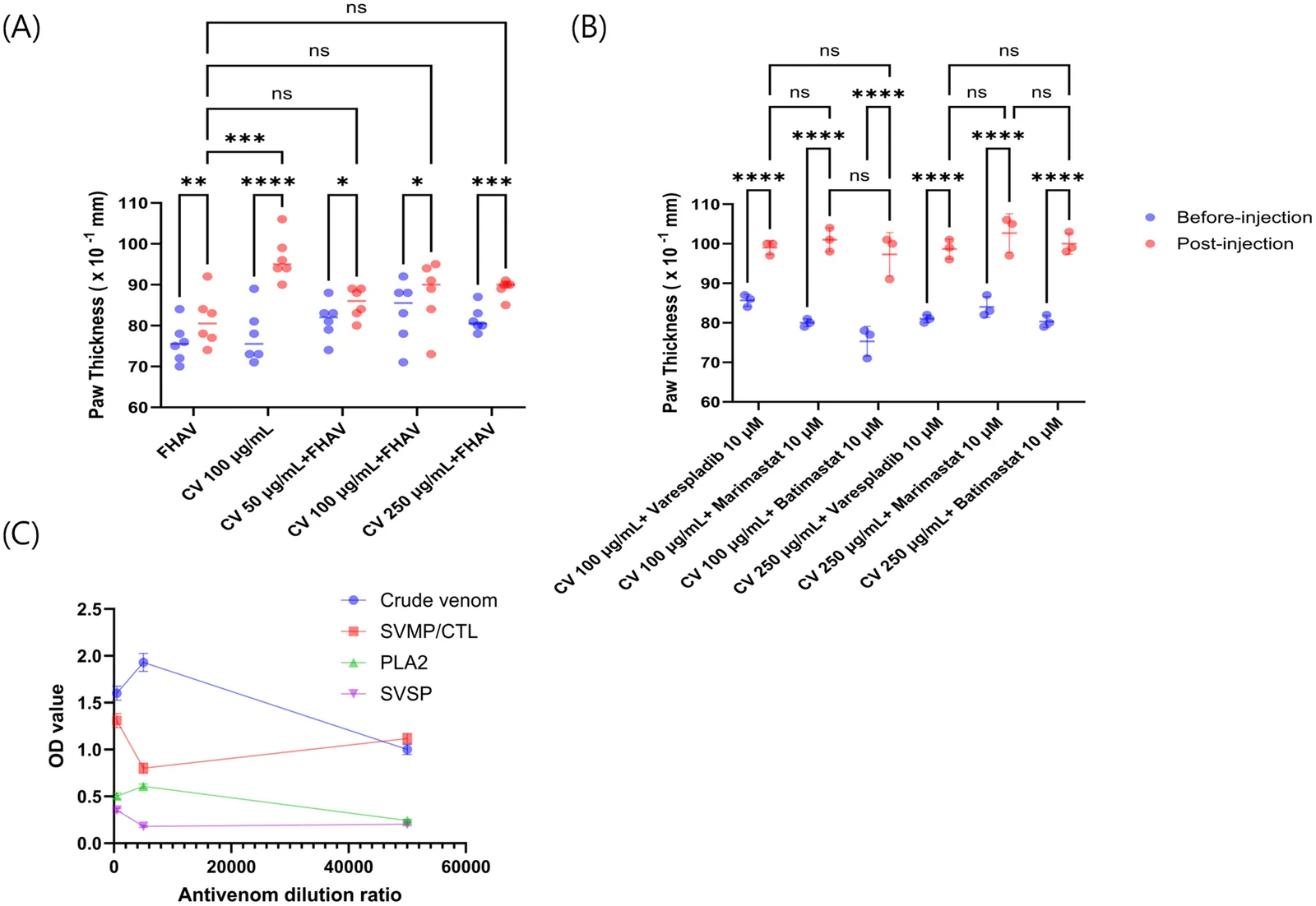
Neutralization of *Protobothrops mucrosquamatus* venom-induced edema with FHAV and small-molecule inhibitors and characterization of antibody immunoreactivity. **(A)** Neutralizing efficacy of FHAV following preincubation with crude venom (CV; 50, 100, and 250 μg/mL) at 37°C for 30 minutes prior to subplantar injection. A mixed-effects model revealed a highly significant interaction between intervention and time (*F*_6, 35_ = 19.40, *P* < 0.0001), demonstrating that while FHAV significantly attenuated edema at lower venom loads (reducing the response to 100 μg/mL by a mean difference of 0.433 mm; *P* = 0.0077), its neutralization reached a functional plateau at 250 μg/mL, where swelling remained statistically indistinguishable from that of the venom-only control (*P* = 0.9902). **(B)** Evaluation of small-molecule inhibitors (SMIs) revealed that at a fixed concentration of 10 μM, varespladib, marimastat, and batimastat failed to prevent edema induced by 100 or 250 μg/mL CV; despite a significant interaction (F_5, 12_ = 4.787, *P* = 0.0123), all inhibitor treatment groups exhibited severe swelling (*P* < 0.0001 versus baseline) that was statistically indistinguishable from that of unneutralized venom. **(C)** Antigenic immunoreactivity of serial dilutions of FHAV determined via indirect ELISAs (1:500–1:50,000), revealing profound immunological mismatch. The antigen factor accounted for 81.49% of the total variance (F_3, 24_ = 1,119, *P* < 0.0001), with the antibody sequestered predominantly by the SVMP/CTL-enriched fraction, whereas the reactivity toward the primary edematogenic driver, PLA_2_, remained significantly lower across all tested dilutions (*P* < 0.0001). The data are presented as the mean ± standard deviation (*n* = 6 for the CV/FHAV groups; *n* = 3 for the SMI groups).

To translate clinical observations to a preclinical setting, the neutralizing efficacy of FHAV against *P. mucrosquamatus* CV induced edema was evaluated using a murine paw model established to simulate the progressive localized swelling seen in envenomated patients. A mixed-effects statistical analysis revealed a highly significant interaction between intervention (venom concentration and antivenom coadministration) and time (*F*_6, 35_ = 19.40, *P* < 0.0001), demonstrating that the presence of FHAV fundamentally altered the temporal trajectory of venom-induced swelling (Fig 2A). CV alone induced rapid and significant increases in paw volume across all tested concentrations, with mean differences in volume before and after exposure measuring 1.467 mm at 50 μg/mL, 1.900 mm at 100 μg/mL, and 1.900 mm at 250 μg/mL (*P* < 0.0001 for all). Preincubation of venom and FHAV significantly attenuated the swelling induced by low to moderate venom loads, reducing the difference in mean swelling from 1.467 mm to 0.4167 mm (*P* = 0.0101) at 50 μg/mL and from 1.900 mm to 0.4333 mm (*P* = 0.0077) at 100 μg/mL. However, at the highest venom concentration (250 μg/mL), antivenom neutralization plateaued; however, although the difference in swelling was partially reduced to 0.767 mm (*P* < 0.0001), the final absolute paw volume in the neutralized group (mean = 8.917 mm) was not significantly different from that in the unneutralized control receiving 250 μg/mL venom alone (mean = 9.167 mm, *P* = 0.9902). These findings demonstrate that while FHAV has a substantial ability to neutralize venom-induced edema, this protective effect is strictly dose dependent. As the concentration of *P. mucrosquamatus* venom increases, the neutralizing demand of a fixed dose of FHAV increases until its protective effect is overwhelmed, statistically confirming a clear dose‒response relationship and suggesting that clinical scenarios involving heavy venom burdens or progressive swelling require increased FHAV doses to achieve complete therapeutic neutralization.

### Limited efficacy of small-molecule inhibitors in neutralizing *Protobothrops mucrosquamatus* venom-induced edema in a murine model

Following the evaluation of swelling induced by individual toxins in the paw edema model, SMIs were used to target specific toxin families: varespladib was used to inhibit PLA_2_, and marimastat or batimastat was used to inhibit SVMP. The three SMIs were prepared at a fixed concentration of 10 μM, preincubated with CV at 100 or 250 μg/mL, and administered via subplantar injection into the hind paw of the mice, after which the paw thickness was measured at 3 h postinjection for comparison with baseline values. The mixed-effects model analysis demonstrated a highly significant main effect of time on paw thickness (*F*_1, 12_ = 1,071, *P* < 0.0001) and a significant interaction between intervention and time (F_5, 12_ = 4.787, *P* = 0.0123). Despite the administration of the SMIs at a concentration of 10 μM, subplantar injection of CV at 100 or 250 μg/mL induced intense and statistically significant local edema (Fig 2B). In the 100 μg/mL CV groups, the mean paw thickness increased significantly from baseline in the varespladib group (8.567 mm to 9.900 mm; *q* = 13.46, *P* < 0.0001), the marimastat group (8.000 mm to 10.010 mm; *q* = 21.20, *P* < 0.0001), and the batimastat group (7.533 mm to 9.733 mm; *q* = 22.10, *P* < 0.0001). Comparisons among the different SMIs during the postinjection phase revealed no statistically significant differences in neutralizing efficacy. After injection of 100 μg/mL CV, there were no significant differences between varespladib and marimastat (*q* = 1.155, *P* = 0.9616) or between marimastat and batimastat (*q* = 2.117, *P* = 0.6694). Similarly, at the higher CV dose of 250 μg/mL, no inhibitor demonstrated superiority with no significant difference detected between marimastat and batimastat (*q* = 1.540, *P* = 0.8811) or between varespladib and batimastat (*q* = 0.7698, *P* = 0.9936). These results indicate that at the tested concentration of 10 μM, none of these three SMIs exhibited significant neutralizing efficacy against the edematogenic activity of CV. This suggests that a single concentration of these monotargeted inhibitors may be insufficient to disrupt the complex toxic pathways in *P. mucrosquamatus* venom or that completely arresting the progressive localized swelling may require higher inhibitor dosages or multimodal targeting rather than single toxin family intervention under these specific experimental conditions.

### Evaluation of the relative immunoreactivity of freeze-dried hemorrhagic antivenom toward crude venom and isolated toxin fractions

An indirect enzyme-linked immunosorbent assay (ELISA) was used to determine the relative immunoreactivity of FHAV toward the CV and purified fractions, including the PLA_2_, SVSP, and SVMP/CTL fractions (Fig 2C). These specific toxin antigens were diluted to a final concentration of 10 µg/mL, various dilutions of FHAV were applied, and the optical density (OD) was subsequently measured to evaluate the binding affinity of FHAV for the different antigens, including CV, ${\text{PLA}}_{\text{2}}$, SVSP, and SVMP/CTL. The immunoreactivity of FHAV for *P. mucrosquamatus* CV and its isolated toxin fractions were assessed using two-way ANOVA. The analysis revealed that antigen type, dilution factor, and their interaction strongly affected antibody binding, with a significant interaction (F_6, 24_ = 85.81; *P* < 0.0001), a main effect of dilution (F_2, 24_ = 112.0; *P* < 0.0001), and a main effect of the antigen (F_3, 24_ = 1,119; *P* < 0.0001). Notably, the antigen accounted for 81.49% of the total variance, indicating a pronounced preference in the FHAV binding profile. Tukey’s *post hoc* multiple comparisons test indicated that at the lowest dilution of 1:500, the binding of FHAV to CV was significantly greater than its binding to any purified fraction, including SVMP/CTL (mean difference: 0.2900, *q* = 9.667, *P* < 0.0001), PLA_2_ (mean difference: 1.097, *q* = 36.56, *P* < 0.0001), and SVSP (mean difference: 1.247, *q* = 41.56, *P* < 0.0001). However, at a greater dilution of 1:50,000, the optical density of CV decreased to a level statistically indistinguishable from that of the SVMP/CTL fraction (mean difference: 0.1167; *q* = 3.889, *P* = 0.0509). Furthermore, the binding of FHAV to SVMP/CTL remained significantly greater than its binding to PLA_2_ and SVSP across all the tested dilutions; for example, at a dilution of 1:50,000, the comparison between SVMP/CTL and PLA_2_ yielded a mean difference of 0.8733 (*q* = 29.11, *P* < 0.0001). Taken together, these findings indicate that while FHAV exhibits immunoreactivity with multiple venom components, its primary immunoreactivity is directed toward the SVMP/CTL fraction. At high dilutions, the recognition of SVMP/CTL is comparable to that of CV, whereas FHAV binding to PLA_2_ and SVSP remains consistently and significantly lower. These results suggest that proteins within the SVMP/CTL family are the predominant antigens recognized by FHAV.

## Discussion

Envenomation by *P. mucrosquamatus* is the leading cause of snakebite-related morbidity in Taiwan and is characterized by debilitating local tissue pathologies such as extensive edema, ecchymosis, and necrosis. While FHAV can successfully manage systemic toxicity, its ability to stop progressive localized swelling remains a subject of clinical concern. This study investigated this discrepancy, predicated on the hypothesis that localized edema is a synergistic pathological response mediated by multiple toxin families, including PLA_2_, SVSP and SVMP/CTL, which exhibit differential susceptibility to neutralization by antivenom. By employing a murine model, to characterize the dose–response relationships and RP-HPLC for venom fractionation, we assessed the relative immunoreactivity and neutralizing efficacy of FHAV and the potential of small-molecule inhibitors as therapeutic adjuncts.

In a clinical cohort of 50 *P. mucrosquamatus*-envenomed patients (Table 1), hospital admission duration was positively associated with recovery duration (*β* = 0.355, T = 3.274, *P* = 0.002), which is consistent with the clinical rationale that more severe tissue injury warrants prolonged inpatient observation. Interpreting clinical recovery in this cohort requires a clear distinction between univariate association and multivariable prediction. With respect to delay in arriving at the hospital, the time elapsed from bite to arrival at the emergency department (bite-to-ED time) averaged 3.17 ± 5.47 h (range: 0–29 h) and was not significantly associated with edema remission duration according to univariate analysis (*r* = -0.029, *P* = 0.422) or as an independent predictor according to a comprehensive multivariable regression model adjusted for 12 clinical variables (*β* = 0.114, T = 1.017, *P* = 0.316). Conversely, initial severity upon emergency department presentation did not significantly correlate with remission duration according to univariate analysis (*r* = 0.093, *P* = 0.061) but emerged as a significant negative independent predictor according to multiple linear regression (*β* = - 0.283, *T* = -2.039, *P* = 0.049). Rather than implying that greater initial severity inherently accelerates recovery, this paradoxical inverse relationship reflects the powerful mediating effect of total FHAV dosage, which served as the primary driver of recovery duration (*β* = 0.658, *T* = 5.167, *P* < 0.001). Because antivenom administration dynamically increased with increasing clinical progression from the initial to final Blaylock grades (*r* = 0.375; *P* = 0.004), the total FHAV dosage strongly correlated with the overall recovery time (*r* = 0.743; *P* < 0.001). Clinically, increased antivenom requirements represent an integrated marker of ongoing venom burden and inflammatory progression, establishing a cumulative FHAV dosage as a more comprehensive indicator of overall disease trajectory and subsequent tissue repair requirements than a static, single-point initial assessment.

The murine paw edema model effectively simulates the progressive localized swelling observed in patients with *P. mucrosquamatus* envenomation (Fig 1A). Published toxicological evaluations involving manual venom gland extraction have indicated that the dried venom yield per adult snake ranges from 6.6 mg to 125.0 mg, with a mean yield of 33.4 mg [31]. In contrast, in our murine subplantar paw edema assay, we evaluated the effects of a fixed volume of 50 μL containing 5 μg of CV or isolated toxin fractions, a concentration range specifically calibrated to elicit robust, quantifiable, and nonlethal localized tissue swelling. Direct linear scaling of human venom delivery loads to rodents is unfeasible because administering venom doses in the milligram range directly into mice would induce immediate, fatal systemic toxicity long before localized edema could be systematically quantified. Although constrained by these inherent physiological scale differences, this murine model successfully captures the kinetic profile of venom-induced tissue damage, providing a reliable and physiologically relevant preclinical platform for evaluating localized edema and assessing the therapeutic neutralization potential of FHAV and SMIs.

Experimental analysis using a mixed-effects model confirmed that FHAV effectively neutralized *P. mucrosquamatus* venom-induced edema (Fig 2A). While CV alone elicited substantial paw swelling, preincubation with FHAV significantly attenuated these responses; for instance, at a venom concentration of 100 μg/mL, the difference in swelling markedly decreased to 0.433 mm (*P* = 0.0077). A clear dose‒response relationship was detected, with the neutralizing capacity of FHAV approaching functional saturation as the venom load increased; however, at 250 μg/mL, although swelling was partially inhibited (the difference decreased to 0.7667 mm), the posttreatment mean of 8.917 mm was not significantly different from that of 9.167 mm in the venom-only group (*P* = 0.9902). Neutralizing local edema induced by *P. mucrosquamatus* venom is a dose-dependent process wherein higher venom loads necessitate increased antivenom dosing to achieve clinical remission. Administered intravenously to ensure immediate systemic bioavailability [32], FHAV is an equine-derived F(ab’)_2_ fragment formulation [21]. Pharmacokinetically, FHAV has a maximum plasma concentration (*Tmax*) of 36 min [32], an initial distribution half-life (*t*_*1/2α*_) of 15 min [33], a terminal elimination half-life (*t*_1/2β_) of 48–96 h [34], and a mean residence time (MRT) of 10.4 d [33]. This prolonged MRT facilitates the sustained neutralization of tissue-released toxins, explaining the lower incidence of rebound envenomation with these formulations than with Fab-based formulations that are rapidly cleared [22]. Consequently, increasing FHAV dosages in patients experiencing progressive swelling represents a statistically supported and mechanistically rational therapeutic strategy to overcome the functional redundancy of venom and achieve comprehensive clinical neutralization. At our institution, the treatment protocol for repeat FHAV administration relies on a point-of-care ultrasound protocol [35], specifically incorporating the Sonographic Assessment of the Rate of Proximal Progression to guide clinicians in administering supplemental FHAV doses [24]. Clinically, continued progression of edema signals an unneutralized venom burden rather than pharmacokinetic failure, indicating the need for continuous dose escalation until swelling expansion demonstrably decelerates, with ultrasonographic monitoring guiding the definitive therapeutic endpoint [24].

A key observation of this study is that none of the three SMIs, namely varespladib, marimastat, and batimastat, produced statistically significant attenuation of *P. mucrosquamatus* venom-induced paw edema under the tested exploratory concentration of 10 μM (*P* < 0.0001; Fig 2B). This screening threshold of 10 μM was systematically selected as a baseline on the basis of established *in vitro* functional profiles, where low-micromolar concentrations up to 10 μM are widely utilized in primary high-throughput screens to capture potent lead molecules while maintaining a robust assay window [36,37]. This concentration serves as a strategic exploratory ceiling given that benchmark metalloproteinase inhibitors such as marimastat and batimastat display half-maximal effective concentrations (EC_50_) in the low-nanomolar range (2.36 nM to 42.0 nM), ensuring near 100% target enzyme inhibition [36,37]. Similarly, although varespladib specifically targets PLA_2_ rather than SVMPs, its reported potency (IC_50_ values of 0.221 μM to 0.276 μM) further supports 10 μM as a rational screening benchmark [36]. Crucially, these initial findings should not be construed as intrinsic insusceptibility of *P. mucrosquamatus* venom to small-molecule therapy, particularly given the extensive literature highlighting SMIs, notably the broad-spectrum PLA_2_ inhibitor varespladib (LY315920), as highly effective candidates capable of neutralizing diverse viperid PLA_2_ isoforms [38,39]. In *P. mucrosquamatus* venom, PLA_2_ variants include catalytically active D49-PLA_2_ (42.27%) alongside enzymatically inactive K49-PLA_2_ (1.58%) and R49-PLA_2_ (56.15%) [5], all of which varespladib neutralizes either by directly blocking the catalytic active site and Ca^2+^-binding loop or by physically occluding the conserved hydrophobic channel [14,40,41]. Because the current evaluation was restricted to a single 10 μM dose without *in vivo* dose titration, these observations likely indicate that 10 μM falls below the local tissue threshold required to fully suppress the synergistic edematogenic cascade driven by PLA_2_, SVSP, and SVMP toxins. Consequently, systematic *in vitro* enzymatic assays, *in vivo* dose-escalation studies, and combination strategies with FHAV remain necessary to rigorously establish the therapeutic utility of SMIs against *P. mucrosquamatus* envenomation.

A critical finding of this study is that FHAV (Taiwan Centers for Disease Control, Taipei, Taiwan) exhibits significantly lower immunoreactivity toward PLA_2_ than toward SVMP/CTL (Fig 2C), a phenomenon consistent with prior toxicological research [16]. Analysis of currently available FHAV formulations demonstrates that the immunogenicity of *P. mucrosquamatus* venom components varies dramatically in close correlation with their respective molecular weights [16]. High-molecular-weight proteins, particularly P-III SVMPs, LAAOs, and serine proteinases, act as dominant immunogens that stimulate robust equine antibody responses due to their large size and structural complexity [16]. In contrast, low-molecular-weight toxins, most notably the neurotoxic D49-PLA_2_ variant, display markedly weak immunogenicity because their smaller dimensions restrict functional epitope exposure [16]. This deficiency is further exacerbated during the immunization process by an antigenic masking or immunosuppressive effect, wherein immunodominant high-molecular-weight SVMPs and SVSPs overshadow smaller toxins, leading to low-titer antibody generation in poor-responding horses [16]. Consequently, the disproportionately weak equine immune response toward low-molecular-weight PLA_2_ enzymes remains the primary driver of the clinical neutralization gaps observed with Taiwan’s current FHAV therapy [16].

To establish a robust translational foundation for optimizing *P. mucrosquamatus* envenomation therapy, four strategic research directions are proposed. First, comprehensive toxicovenomic mapping and comparative potency profiling via RP-HPLC across Taiwan are required to resolve spatial heterogeneity, particularly inter-population variations in acidic PLA_2_ and SVMP abundance between northern and southeastern regions. Second, preclinical evaluations must advance beyond static 10 μM screening by implementing systematic dose-escalation protocols, precise venom-to-inhibitor molar-ratio calculations, and subplantar paw edema models to establish half-maximal inhibitory concentrations (IC_50_) and median effective doses (ED_50_). These assays should prioritize multimodal synergistic regimens, including SMI-SMI cocktails and SMI combinations with FHAV (Taiwan Centers for Disease Control, Taipei, Taiwan), to resolve the severe immunological mismatch wherein 81.49% (*P* < 0.0001) of FHAV binding is sequestered by the SVMP/CTL fraction, leaving highly edematogenic PLA_2_ and SVSP toxins inadequately neutralized. Third, because indirect ELISA reflects antigen recognition rather than functional neutralization, future work must establish toxin-specific functional assays and complete dose-response curves to determine true neutralization efficiencies and verify whether reduced immunoreactivity directly drives persistent clinical edema. Fourth, experimental design must transition from pre-incubation models to post-envenomation rescue protocols incorporating delayed-treatment timelines, accounting for the rapid subcutaneous diffusion of PLA_2_ and the extravasation kinetics of antivenom F(ab’)_2_ fragments within swollen interstitial spaces. Ultimately, these integrated preclinical insights will inform prospective multicenter clinical trials to transition patient management from empirical dosing to standardized, potency-aligned FHAV therapeutic protocols.

### Limitations

Several methodological limitations inherent to the design of this investigation warrant consideration across its sequential stages. Regarding the clinical cohort, the retrospective sample size was relatively small (*N* = 50) and consisted primarily of mild-to-moderate envenomations categorized via the Blaylock classification [28] rather than the Snakebite Severity Score [42] to prioritize localized edema, with resolution timelines relying on patient self-reporting subject to recall bias in the absence of objective sonographic or functional measurements. Regarding venom sampling and dosing, CV derived from a single source failed to capture regional toxicological diversity across Taiwan, while fixed experimental doses (2.5 μg and 5 μg for PLA_2_; 12.5 μg and 25 μg for SVSP and SVMP/CTL in 50 μL) evaluated intrinsic potency on an equal protein mass basis rather than mirroring natural proteomic proportions (PLA_2_ constituting 15.9%-25% of CV), thus complicating direct translational extrapolation [31]. Regarding the animal model, although the rodent paw assay demonstrated high statistical sensitivity (*F*_1, 35_ = 3,490; *P* < 0.0001), mice remain an imperfect proxy for human subcutaneous circulation kinetics, and the exclusive use of male ICR/CD-1 mice (*n* = 6 per group) to eliminate estrous variability left the study unpowered to assess sex-specific differences despite female victims accounting for 32% of the clinical cohort. Regarding therapeutic evaluations, antivenom neutralization was assessed using a single lot of FHAV (lot no. 60-06-0020; Taiwan Centers for Disease Control, Taipei, Taiwan), precluding evaluation of lot-to-lot variability. Finally, pooled fractions were assigned based on established chromatographic profiles [6] without independent reconfirmation by LC-MS/MS, SDS-PAGE, or enzymatic assays, indicating that these preparations represent toxin family-enriched fractions that may contain coeluting proteins.

## Conclusions

In conclusion, this study identifies the PLA_2_-enriched fraction as the primary driver of rapid, high magnitude edema after *Protobothrops mucrosquamatus* envenomation, as it elicits marked swelling at substantially lower protein concentrations than the SVSP-enriched or SVMP/CTL-enriched fractions do. Despite its critical role, a profound “immunological mismatch” exists wherein 81.49% of FHAV immunoreactivity is sequestered by the SVMP/CTL-enriched fraction, leaving the highly edematogenic PLA_2_ component poorly targeted. Currently, increasing FHAV dosages in patients with progressive swelling remains the most viable clinical strategy to compensate for this low specific immunoreactivity through increased total antibody volume. It is essential to recognize that the positive correlation between total FHAV dosage and the remission timeline represents a clinical association rather than direct causation, as higher dosages are typically a reactive integrated marker of the ongoing venom burden. Furthermore, the limited efficacy of SMIs at 10 μM suggests that single-target interventions are insufficient to disrupt the functional redundancy of the venom. Ultimately, robust prospective multicenter trials are needed to transition from empirical dosing to standardized, potency-aligned FHAV protocols to ensure comprehensive neutralization of all major toxin families.

**Declaration of the Use of AI and Language Editing:** During the preparation of this work, we utilized Google Gemini and NotebookLM to refine the language and improve the logical flow of the initial draft. Following this refinement, we reviewed and edited the content as necessary and assume full responsibility for the integrity of the final publication, with subsequent professional English language editing and certification services provided by American Journal Experts.
