# Supplementary material for: Why does local edema persist? Immunological mismatch between phospholipase A2 potency and antivenom affinity in Protobothrops mucrosquamatus envenomation
Source: PLoS Negl Trop Dis. 2026 Sep 3;20(9):e0014689. doi: 10.1371/journal.pntd.0014689 (PMC13541122; doi:10.1371/journal.pntd.0014689)
Supplement: Supplementary file 1 [file pntd.0014689.s002.docx]

Aug 12, 2026

On behalf of all the authors

Editor-in-Chief

PLOS Neglected Tropical Diseases

Dear Editorial Board Members:

Enclosed is a revised version of the manuscript titled **“Why Does Local Edema Persist? Immunological Mismatch between Phospholipase A2 Potency and Antivenom Affinity after *Protobothrops mucrosquamatus* Envenomation**" (Manuscript ID: PNTD-D-26-01063). The comments from the reviewers were valuable and helpful in revising our manuscript, and we appreciate the opportunity for resubmission. We have revised the manuscript on the basis of the recommendations and responded to all the questions raised by the reviewers, including providing point-by-point responses to the comments. Thank you for considering our revised manuscript.

Sincerely,

Cheng-Hsuan Ho, MD, PhD

Attending Physician

Department of Emergency Medicine, Tri-Service General Hospital, National Defense Medical University, Taipei City 114202, Taiwan No. 325, Sec. 2, Cheng-Kung Road, Neihu Dist., Taipei City 11490, Taiwan Tel.: +886-2-87923311-16877 Fax: +886-2-87927034 E-mail: erdoctorho@gmail.com

E-mail: [erdoctorho@gmail.com](mailto:erdoctorho@gmail.com)

# Reviewer 1

- **In the Results section (lines 330–332), the authors report that while P. mucrosquamatus venom induces potent, dose-dependent swelling, freeze-dried horse antivenom (FHAV) provides effective neutralization of this edematogenic activity. Conversely, the authors pinpoint the limited efficacy of a single dose of varespladib, marimastat, and batimastat in resolving this edema. While this limitation is appropriately addressed in the Discussion, it should be explicitly mentioned in the Results section as well. It has long been established that antivenom dosing must be titrated against the severity of a patient's clinical presentation. Therefore, while these results are original, they are not entirely unexpected. It is highly probable that small-molecule inhibitors would follow the same dose-response principles (i.e., requiring dose escalation corresponding to venom load).**

Ans: We thank the reviewer for this insightful comment regarding the presentation of our small-molecule inhibitor data and the fundamental principles of dose titration. We agree that small-molecule inhibitors, such as varespladib, marimastat, and batimastat, likely follow classic dose-response kinetics, where dose escalation relative to the initial venom load is required to achieve functional neutralization, mirroring the established clinical titration of antivenom. In accordance with your recommendation, we have explicitly incorporated this limitation into the Results section of the revised manuscript, clarifying that the tested concentration of 10 μM represents a single exploratory baseline rather than a maximal therapeutic ceiling, as now reflected at lines 539 to 549 (highlighted in blue text) of the revised manuscript.

- **The authors highlight the immunological mismatch between toxin potency and antivenom binding specificity as their most significant finding. However, this phenomenon is relatively predictable. The dilutional effect of therapeutic IgG caused by highly abundant, immunodominant (but less toxic) antigens is a well-known hurdle in antivenom therapy. The authors should contextualize this finding more nuances in the text to reflect existing literature on antivenom immunogenicity.**

Ans: We thank the reviewer for this constructive insight into the well-established immunodynamics of antivenom production driven by antigen abundance and structural complexity. In accordance with your recommendation, we have thoroughly updated the Discussion section to contextualize our findings within the broader literature on *Protobothrops mucrosquamatus* venom immunogenicity. Specifically, we explicitly address how high-molecular-weight toxins, such as P-III SVMPs and snake venom serine proteases, act as immunodominant antigens that elicit robust antibody responses owing to their structural complexity. Conversely, low-molecular-weight proteins display lower immunogenicity owing to restricted epitope exposure. We emphasize that the primary driver of clinical neutralization gaps observed with FHAV lies in its weak neutralization of PLA₂, particularly the neurotoxic D49-PLA₂ variant. The failure of immunizing horses to generate high-titer antibodies against D49-PLA₂ is likely exacerbated by an antigenic masking effect during equine immunization, wherein larger, highly immunogenic proteins overshadow smaller, less abundant toxins, as now reflected at lines 565 to 581 (highlighted in blue text) of the revised manuscript.

- **The authors’ conclusion and recommendation that “robust prospective multicenter trials are needed to transition from empirical dosing to standardized, potency-aligned FHAV protocols” is excellent and should be strongly emphasized in the final abstract and conclusion sections.**

Ans: We deeply appreciate the reviewer’s positive evaluation of our recommendation. In accordance with this valuable suggestion, we have strongly emphasized the necessity of robust prospective multicenter trials in both the Abstract and Conclusion sections of the revised manuscript, highlighting their critical role in transitioning from empirical dosing to standardized, potency-aligned FHAV protocols.

- **Please use standard mathematical and scientific notation throughout the Methods section rather than writing units out in text. Examples: Use -20°C instead of "minus 20 degrees Celsius"; 0.1% instead of "0.1 percent"; and 20 ± 2 g instead of "20 plus or minus 2 grams".**

Ans: We thank the reviewer for this constructive suggestion regarding formatting consistency. In accordance with your recommendation, we have thoroughly revised the Materials and Methods section to replace all textual representations of units and mathematical operators with standard scientific and mathematical notation, including expressions such as -20 °C, 0.1%, and 20 ± 2 g, as now reflected throughout the Materials and Methods section (highlighted in blue text) of the revised manuscript.

- **Standardize the notation for critical reagents and manufacturers. Use brackets to denote locations and streamline the text. Example: Use Gibco (Thermo Fisher Scientific, Waltham, MA, USA) instead of "Gibco at Thermo Fisher Scientific in Waltham, Massachusetts, USA".**

Ans: We thank the reviewer for this constructive suggestion regarding reagent standardization. In accordance with this recommendation, we have thoroughly updated the Materials and Methods section (highlighted in blue text) to standardize the formatting for all critical reagents, kits, and equipment by utilizing bracketed manufacturer information, including city, state, and country details, such as that of Gibco (Thermo Fisher Scientific, Waltham, MA, USA), ensuring uniform presentation throughout the text.

- **Remove the duplicated section title found in lines 207–208: "Characterization of Antivenom Binding Profiles via Indirect Enzyme Linked Immunosorbent Assay".**

Ans: We thank the reviewer for pointing out this editorial oversight. In accordance with the reviewer's suggestion, we have removed the duplicate section title from the manuscript, as reflected in lines 232 of the revised version.

- **Ensure that Latin terms such as in vivo are consistently italicized throughout the entire manuscript.**

Ans: We thank the reviewer for this helpful comment. In accordance with your recommendation, we have thoroughly reviewed the entire manuscript and ensured that all Latin terms, such as *in vivo* and *in vitro*, are consistently italicized throughout, as now reflected throughout the revised manuscript (highlighted in blue text).

# Reviewer 2

- Objectives and study design are clearly articulated. Sample sizes and statistics are appropriately described. No ethical or regulatory concerns.
- Results are clear, images need to be of higher resolution for publication.
- Conclusions are generally supported, but some claims are too bold. Details are provided below (in the summary and comments section).
  Limitations are described. The authors discuss their findings in the context of shaping clinical care.
- Some panels in a couple of figures are superfluous (details provided below).
- In this study, the authors aim to understand the underlying causes of persistent edema in patients envenomed with Protobothrops microsquamatus venom despite administration of standard antivenom. They investigate this in a mouse model of edema and fractionate the venom to identify the toxins responsible for this outcome. They note that toxins resulting in local edema are not effectively neutralised by the current antivenom regimen and recommend increasing antivenom doses in parallel with developing antivenoms that better neutralise these toxins (SVSPs and PLA2s). The study is well conducted and logically presented but could benefit from a more extensive setting up of the experimental questions before delving into the data. Some conclusions are also quite strongly presented and require further discussion.
- **Lines 272- paragraph starts with conclusions but the experimental setup/ hypotheses are not described. Please insert a short paragraph setting up the question and experiment.**

Ans: We thank the reviewer for this constructive suggestion regarding the logical structure of our manuscript. In accordance with your recommendation, we have added a concise introductory paragraph immediately preceding the Results section at lines 309 to 312 (highlighted in blue text) of the revised manuscript. This addition explicitly articulates our core toxicological hypothesis and experimental setup, thereby providing an essential conceptual framework for our subsequent findings.

- **Figure 1A – I am uncertain whether figure 1A is needed as the changes at 1-2.5 mm are too small to be visualised by the reader on the ruler, especially at a distance. In addition, the variability in picking the location of the measurements (some at 11 cm, some at 13 or 17 cm) is confusing. I would suggest to just retain figure 1B.
  Figure 1B is also fuzzy and of low quality. Please increase the resolution of all figures in the manuscript.**

Ans: We thank the reviewer for this constructive recommendation to enhance the visual clarity and overall presentation of our figures. In accordance with your advice, we have completely removed the original Figure 1A to eliminate potential confusion surrounding subtle millimeter-scale changes and variable measurement locations on the ruler, retaining Figure 1B and reordering the figure sequence accordingly. Furthermore, all figure panels throughout the manuscript have been replaced with new, high-resolution image files generated at 1200 dpi to ensure optimal graphical legibility and strictly comply with the publication standards of the journal.

- **Question: How much venom is there likely to be delivered in a human and how does this scale with the edematogenic mouse model?**

Ans: We thank the reviewer for raising this important question regarding the translational scaling of venom dosage between human envenomation and our mouse model. We acknowledge that the exact mass of venom delivered during an actual *Protobothrops mucrosquamatus* bite in humans cannot be measured or controlled in real time. Published toxicological studies involving manual venom gland extraction have indicated that the dry venom yield per adult snake ranges from 6.6 mg to 125.0 mg, with a mean yield of 33.4 mg. In contrast, our murine subplantar paw edema assay evaluated a fixed volume of 50 μL containing 5 μg of crude venom or isolated toxin fractions, doses specifically calibrated to elicit robust, quantifiable, and nonlethal localized swelling without inducing mortality. Direct linear scaling from human venom yields to rodents is unfeasible because administering milligram-scale venom doses to mice would induce rapid, fatal systemic toxicity long before localized tissue edema could be systematically quantified. As detailed at lines 492 to 505 (highlighted in blue text) of the revised manuscript, this sublethal dose model isolates acute local edematogenic dynamics while eliminating systemic confounding factors.

- **Section lines 289-291. As above, this section needs an introductory paragraph stating the hypothesis behind the experiments and describing the experimental setup – for example: ‘To determine the constituents of the venom, we performed reverse phase HPLC…’ etc**

Ans: We thank the reviewer for this constructive suggestion to enhance the transitional clarity of our manuscript. In accordance with your recommendation, we have added a concise introductory paragraph at the beginning of the venom characterization subsection in lines 332 to 335 (highlighted in blue text) of the revised manuscript. This addition explicitly articulates our underlying toxicological hypothesis and outlines the experimental setup, including reverse-phase high-performance liquid chromatography analysis, thereby providing essential contextual framing for the analytical findings that follow.

- **Line 297 – section on the mouse paw edema model. A sentence on how the doses and administration of the fractions in the mouse model were decided is needed. How were the doses chosen – based on toxin abundance/ fraction of the total venom in line with previous doses? The rationale behind this is missing. Are the doses for which you see a marked effect in the assay relevant in a patient following envenomation? Please discuss (for example you used 5 ug of PLA2; how much would you normally expect in venom percentage wise and how much would be delivered in a bite?)**

Ans: We thank the reviewer for this insightful critique regarding the rationale for selecting specific toxin dosages and their clinical relevance in our murine paw edema model. We wish to clarify that the primary objective of this *in vivo* assay was to compare the relative intrinsic edematogenic potency of major isolated toxin fractions on a standardized protein mass basis rather than to replicate the exact proportional composition of crude venom delivered during natural envenomation. Each fraction was administered in a fixed volume of 50 μL, where the PLA₂ fraction was evaluated at concentrations of 50 μg/mL and 100 μg/mL (corresponding to absolute doses of 2.5 μg and 5.0 μg per paw, respectively), while the SVSP and SVMP/CTL fractions were evaluated at 250 μg/mL and 500 μg/mL (corresponding to 12.5 μg and 25.0 μg per paw, respectively). These dose ranges were established via preliminary evaluations to elicit robust, quantifiable, and nonlethal localized swelling comparable to the inflammatory response induced by 5.0 μg of crude venom, with higher concentrations for SVSP and SVMP/CTL reflecting their lower baseline edematogenic activity. Proteomic studies indicate that PLA₂ constitutes approximately 15.9% to 25.0% of *Protobothrops mucrosquamatus* venom, meaning that 5.0 μg of isolated PLA₂ compositionally corresponds to approximately 20.0 μg to 31.0 μg of crude venom. Direct translational scaling from these murine doses to human clinical scenarios remains highly challenging because human bites deliver variable payloads averaging 33.4 mg and reaching 125.0 mg, doses that would induce immediate systemic lethality in rodents before localized edema could be systematically quantified. As detailed at lines 492 to 505 and 614 to 620 (highlighted in blue text) of the revised manuscript, this sublethal model isolates acute local edematogenic kinetics, providing a reliable preclinical platform to evaluate tissue swelling and assess the therapeutic neutralization potential of FHAV and SMIs.

- **Fig 2 – same as above please increase figure resolution**

Ans: We thank the reviewer for this constructive recommendation to improve the visual quality and clarity of our illustrations. We have replaced all remaining figure panels throughout the manuscript with new, high-resolution image files prepared at 1200 dpi to guarantee optimal graphical legibility and strictly comply with the publication standards of the journal.

- **Lines – 317 – Please lead with the question and what is being tested and how – describe venom doses, administration, AV doses, preincubation. Would these doses be relevant in a real life bite scenario?**

Ans: We thank the reviewer for this constructive request to clarify our research question, experimental design, and clinical translation. To explicitly address what was tested and how, our *in vivo* study evaluated the neutralizing efficacy of FHAV and SMIs against localized tissue swelling induced by *Protobothrops mucrosquamatus* venom, wherein mice received a 50 μL subplantar injection containing 5.0 μg of crude venom pre-incubated with specified doses of antivenom or inhibitors, alongside post-envenomation rescue protocols. In terms of translational relevance in a real-life bite scenario, published toxicological evaluations indicate that dry venom yields per adult *P. mucrosquamatus* range from 6.6 mg to 125.0 mg, with a mean yield of 33.4 mg. In contrast, our murine assay evaluated a fixed volume of 50 μL containing 5.0 μg of venom or toxin fractions, a sublethal concentration specifically calibrated to elicit robust, quantifiable, and nonlethal localized swelling. Direct linear scaling of human venom payloads to rodents is unfeasible because administering milligram-scale doses to mice would induce immediate, fatal systemic toxicity long before localized edema could be systematically quantified. Although constrained by these inherent physiological scale differences, this sublethal model captures acute localized swelling kinetics, providing a reliable preclinical platform to evaluate therapeutic neutralization. Furthermore, to align preclinical methodologies with institutional clinical protocols, where ongoing edema progression reflects an unneutralized venom burden requiring point-of-care ultrasound-guided repeat antivenom administration, research must transition from pre-incubation assays to delayed post-envenomation rescue models that account for the rapid subcutaneous diffusion of PLA₂ and the extravasation kinetics of antivenom F(ab')₂ fragments within swollen interstitial spaces, as now thoroughly detailed at lines 364 to 367 and lines 492 to 505 (highlighted in blue text) of the revised manuscript.

- **Line 324 – I recommend changing ‘co-administration’ to ‘the preincubation of venom and antivenom…’ , as co-administration could be interpreted as given at the same time but without a preincubation step.**

ANS: We thank the reviewer for this insightful clarification regarding the distinction between simultaneous administration and prior mixing. In accordance with your recommendation, we have updated our terminology throughout the manuscript to replace “co-administration” with “pre-incubation of venom and antivenom.” Specifically, we revised the manuscript text to state that the pre-incubation of crude venom with FHAV successfully attenuated the observed edematogenic effects, as now reflected at line 374 (highlighted in blue text) of the revised manuscript.

- **Fig 3 – as in figure 1, I recommend removing panel A. The images can be included in the supplementary if desired, but panel B is sufficient to make the point presented in A.**

Ans: We thank the reviewer for this constructive recommendation to enhance the visual quality and clarity of our illustrations. In accordance with this advice, we have completely removed the original Figure 3A from the manuscript to eliminate any potential confusion surrounding the subtle measurement variations on the ruler. Additionally, we have replaced all remaining figure panels throughout the manuscript with new, high-resolution image files prepared at 1200 dpi to guarantee optimal graphical legibility and strictly comply with the publication standards of the journal.

- **SMI paragraph (line 334) – as above, please lead with the hypothesis and experimental setup in a few sentences before describing the results**

Ans: We thank the reviewer for this helpful suggestion to clarify the logical foundation of SMI evaluations. In accordance with your recommendation, we have inserted a concise introductory paragraph at the beginning of the corresponding section to explicitly articulate our underlying hypothesis and outline the experimental setup prior to presenting the results, which can now be reviewed at lines 392 to 397 (highlighted in blue text) of the revised manuscript.

- **For the SMI experiments how was the tested 10 uM concentration chosen – were any higher doses used/titrations done?**

Ans: We thank the reviewer for this important question regarding our rationale for the experimental doses of SMI used. The screening concentration of 10 μM was systematically selected as a standardized exploratory ceiling on the basis of established *in vitro* functional profiles in the literature, where low-micromolar concentrations of up to 10 μM are widely utilized for the initial screening of toxin inhibitors. Specifically, Clare et al. successfully employed 10 μM marimastat as a maximal inhibitory control in a high-throughput SVMP assay, establishing this threshold as the required dose to completely suppress enzymatic activity before conducting downstream serial dilutions to subnanomolar ranges. That study also confirmed that both marimastat and batimastat effectively neutralized the activity of SVMPs from various medically significant viper venoms within the low-nanomolar range, thereby validating 10 μM as a robust exploratory ceiling. With respect to varespladib, previous investigations demonstrated clear inhibition of venom PLA₂ activity within the low-micromolar range; for instance, le Roux et al. reported that varespladib significantly attenuated anticoagulant activity at low-micromolar concentrations, whereas high-resolution nanofractionation studies evaluated both varespladib and marimastat at concentrations up to 20 μM during toxin profiling. On the basis of these collective benchmarks, 10 μM was chosen as an exploratory concentration that exceeded the documented effective ranges for multiple PLA₂ and SVMP targets, enabling a rigorous and direct comparison of varespladib, marimastat, and batimastat under identical experimental conditions, as detailed at lines 539 to 549 (highlighted in blue text) and the reference 36, 37 of the revised manuscript. Furthermore, we explicitly acknowledge the limitations of relying on a single fixed concentration and emphasize the necessity of expanded dose-escalation evaluations in future studies, as now reflected at lines 558 to 564 and lines 587 to 595 (highlighted in blue text) of the revised manuscript.

- **Line 354 – This statement is too bold – testing at a single concentration may not be enough to support this conclusion. Please rephrase.**

Ans: We thank the reviewer for pointing out this overstatement regarding our small-molecule inhibitor data, and we completely agree that evaluating these inhibitors at a single concentration of 10 μM is insufficient to draw definitive conclusions regarding their absolute therapeutic efficacy. Accordingly, we have thoroughly revised our statements to tone down our conclusions and avoid overinterpreting these initial single-dose findings, as now reflected at lines 415 to 430 (highlighted in blue text) of the revised manuscript.

- **Line 358 – Please describe what was done – how was the Elisa set up, what was tested**

Ans: We thank the reviewer for this helpful recommendation to provide a detailed description of our enzyme-linked immunosorbent assay (ELISA) protocol prior to describing our statistical analysis. In accordance with your suggestion, we have incorporated this detailed experimental protocol into the Materials and Methods section, as now reflected at lines 424 to 430 (highlighted in blue text) of the revised manuscript.

- **For the discussion on varespladib – lines 428-430 – please consult these papers regarding inhibition of myotoxins and structural insights:**
  [**https://pmc.ncbi.nlm.nih.gov/articles/PMC6868273/**](https://pmc.ncbi.nlm.nih.gov/articles/PMC6868273/)
  [**https://pubmed.ncbi.nlm.nih.gov/36403756/**](https://pubmed.ncbi.nlm.nih.gov/36403756/)

Ans: We thank the reviewer for recommending these two valuable references regarding the structural insights and mechanisms underlying myotoxin inhibition by varespladib. We have thoroughly reviewed the findings of both studies and have integrated their critical perspectives into the Discussion section to further clarify the toxicological context surrounding PLA₂ inhibition. This expanded discussion, along with the corresponding citations, is now reflected at lines 553 to 558 (highlighted in blue text) and in References 40 and 41 of the revised manuscript.

- **Lines 433-435: The statement is too bold. ‘Paradoxically…. SMIs failed to neutralise’ – at the tested doses, a fact which I believe is important, as no titration experiments with various doses of SMIs have been done. Please modify accordingly. In addition, to make the claim that the venom of P. microsquamatus cannot be neutralised by these inhibitors additional in vitro tests assessing the SVMP and PLA2 activity of the venom in the presence or absence of inhibitors would need to be performed and finally, as noted, a titration of SMIs with higher inhibitor doses in the mouse paw edema model would be required as well.**

Ans: We sincerely thank the reviewer for this insightful critique regarding the interpretation of our small-molecule inhibitor data, and we completely agree that asserting absolute therapeutic failure on the basis of a single tested dose of 10 μM represents an overstatement. In accordance with your recommendation, we have thoroughly revised our statement to clarify that the observed lack of neutralization applies strictly to the specific experimental conditions and single concentration evaluated in this study rather than indicating absolute venom insusceptibility. Furthermore, we have expanded the Discussion section to explicitly address these critical limitations, providing a detailed rationale for selecting 10 μM as an initial screening benchmark while emphasizing that comprehensive *in vitro* functional assays evaluating the activities of SVMP and PLA₂, alongside *in vivo* dose escalation studies in the murine paw edema model, are essential to fully elucidate the therapeutic efficacy of these inhibitors, as detailed in lines 536 to 534 (highlighted in blue text) of the revised manuscript.

- **Line 464 – discussion on supplemental antivenom – how does the venom dose vs antivenom dose translate from the murine model to clinical care? In addition, what is the incidence of adverse reactions in patients for this particular antivenom?
  In terms of safety for antivenom administration, when would a second dose of antivenom be recommended? If the MRT is 10 days, is the level of antivenom suboptimal to neutralise remaining toxins?**

Ans: We thank the reviewer for these constructive comments regarding dose translation, antivenom safety profiles, and administration protocols. Regarding dose translation, direct linear scaling from human venom yields (mean of 33.4 mg, range of 6.6 mg to 125.0 mg) to rodents is unfeasible because milligram-scale doses would induce fatal systemic toxicity in mice long before localized edema could be quantified; thus, our subplantar paw edema assay evaluated 5 μg of venom in a volume of 50 μL, a dose specifically calibrated to model localized tissue swelling dynamics and assess the therapeutic neutralization potential of FHAV and small-molecule inhibitors, as now elaborated at lines 492 to 505 (highlighted in blue text) of the revised manuscript. Furthermore, in terms of safety, adverse reactions following FHAV administration remain relatively uncommon, with early mild allergic reactions occurring within 1 hour in approximately 3% of patients, whereas delayed serum sickness typically manifests 4 to 14 days post-administration, with the risk increasing markedly at cumulative doses exceeding 20 vials, as discussed at lines 55 to 61 (highlighted in blue text) of the revised manuscript. Finally, the antivenom administration protocol and MRT considerations have been expanded and can now be reviewed at lines 522 to 535 (highlighted in blue text) of the revised manuscript.

- **Lines 485-487 – Please specify how these tests will be done – for example ‘dose escalation studies in preclinical models, synergistic cocktail testing in vitro and in vivo’. While I agree that marimastat and varespladib have proven preclinical efficacy against multiple venoms, a full body of in vitro evidence and in the murine model would be required P. microsquamatus to support clinical translation for these molecules.**

Ans: We thank the reviewer for this exceptionally constructive suggestion regarding the specific methodological pathways required to support the clinical translation of these therapeutic candidates. To establish a robust scientific foundation for the translation of SMIs and combination therapies against *Protobothrops mucrosquamatus* envenomation, we have significantly expanded our discussion of future research directions at lines 587 to 595 (highlighted in blue text) of the revised manuscript. Specifically, to overcome the limitations of the baseline 10 μM screening concentration, prospective preclinical evaluations must incorporate systematic *in vitro* and *in vivo* dose-escalation assays, precise venom-to-inhibitor molar ratio calculations, and subplantar paw edema models to determine formal half-maximal inhibitory concentration (IC₅₀) and median effective dose (ED₅₀) values. Furthermore, these preclinical evaluations will prioritize multimodal synergistic regimens, such as SMI-SMI cocktails and SMI-FHAV combinations, to resolve the pronounced immunological mismatch and neutralization gaps associated with FHAV.

- **Final paragraph on inhibitors in conclusion (lines 518-521) – It cannot be concluded based on a single dose of inhibitor that the inhibitors have failed without testing higher doses. I recommend removing this rationale from the conclusion.**

Ans: We entirely agree with the reviewer’s judicious assessment. In accordance with your recommendation, we have reframed our conclusion at lines 645 to 647 (highlighted in blue text) of the revised manuscript to clarify that the lack of complete edema suppression at 10 μM must be interpreted cautiously, acknowledging that initial single-dose screening serves as an exploratory baseline rather than a definitive therapeutic ceiling.

- **Line 276 – CV abbreviation was not previously defined**

Ans: We thank the reviewer for this attentive reading and for pointing out the undefined abbreviation. In accordance with your comment, we have explicitly defined crude venom (CV) at its first occurrence at line 197 (highlighted in blue text), and we have ensured that the abbreviation CV is used consistently throughout the remainder of the revised manuscript.

- **Fig. 1 legend lines 17-19. ‘The results show a clear dose-dependent relationship …. This is a sentence that should not be part of the figure legend, but the results section.**

Ans: We thank the reviewer for this helpful comment regarding the formatting of the figure legends. In accordance with your suggestion, we have removed the interpretative statement describing the dose-dependent relationship from the legend of Figure 1 and relocated it to the main Results section. The legend for Figure 1 has been revised to focus strictly on the experimental conditions and visual data, as reflected in the revised manuscript.

- **References 20 and 23 are identical**

Ans: We thank the reviewer for their attentive reading and for identifying duplicate reference entries in our manuscript. We have corrected this clerical error by removing the redundant entry, renumbering the bibliography accordingly, and ensuring that all remaining citations accurately and uniformly correspond to the intended published study. The updated in-text citations and corrected reference entries can now be verified in reference 39 of the revised manuscript.

# Reviewer 3

- **1. Line 19-24. SVMPs cause more than just disruption of vascular integrity, they also degrade the extracellular matrix of muscle cells leading to poor regeneration. So, their effects must be expanded with appropriate references.**

Ans: We thank the reviewer for this excellent recommendation to expand upon the complex pathophysiological roles of SVMPs. We completely agree that in addition to disrupting vascular integrity, these enzymes actively degrade extracellular matrix and basement membrane components of muscle tissue, fundamentally disrupting the cellular microenvironment and impairing successful myofiber regeneration. In accordance with your constructive comment, we have thoroughly expanded the relevant section to delineate these secondary myotoxic mechanisms and integrated key literature references supporting these toxicological actions, as now reflected at lines 20 to 34 (highlighted in blue text) of the revised manuscript.

- **2. Line 81-83. Please provide a reference for support it.**

Ans: We thank the reviewer for this constructive suggestion. In accordance with your recommendation, we have incorporated the appropriate supporting citations (references 29 and 30) to substantiate this statement, which can be verified in line 113 to 115 (highlighted in blue text) of the revised manuscript.

- **3. Line 81. Please replace minus by –**

Ans: We thank the reviewer for this helpful formatting suggestion. In accordance with your recommendation and standard scientific notation guidelines, we have revised the text to replace the written temperature designations with the standard format (-20°C), which can now be verified in lines 112 (highlighted in blue text) of the revised manuscript.

- **4. Lines 91-92. Venom enzymes are usually stable, mainly PLA2 which have 7 disulfide bounds. No loss of activity has been reported at room temperature for venom aliquots.**

Ans: We thank the reviewer for this insightful comment regarding the structural stability and conserved disulfide bonds of snake venom proteins such as PLA₂. Our intention was not to imply rapid inactivation under ambient conditions but rather to explain our protocol of storing single-use aliquots at -20 °C as a standardized, conservative measure to prevent artifactual degradation across all enzymatic constituents and avoid repeated freeze-thaw cycles. In accordance with your recommendation, we have revised the manuscript text at lines 119 to 122 (highlighted in blue text) to remove any statements implying that brief room-temperature exposure inherently causes a loss of biological activity.

- **5. Line 141. Please use symbols to replace the words plus and minus**

Ans: We thank the reviewer for this helpful formatting correction. In accordance with your recommendation and standard scientific notation guidelines, we have replaced the written phrase “plus and minus” with the standard symbol (±), as reflected in the expression “20 ± 2 g” in line 172 (highlighted in blue text) of the revised manuscript.

- **6.** **Line 141. Why do the authors work with male mice? Based on the findings, the authors showed the female are also an important number of snakebite victims.**

Ans: We thank the reviewer for highlighting this important clinical consideration. Male ICR/CD-1 mice (*n* = 6 per group) were selected to maintain strict methodological consistency across all dose range, neutralization, and small-molecule inhibitor assays by eliminating potential confounding physiological variability driven by the murine estrous cycle. While we fully recognize that female patients constitute 32% of the *Protobothrops mucrosquamatus* envenomation cases in our clinical cohort, our preclinical experimental design was not structured or powered to evaluate sex-specific differences in venom-induced edema or therapeutic efficacy. We have explicitly addressed this constraint as a study limitation in lines 622 to 634 (highlighted in blue text) of the revised manuscript.

- **7. Lines 159-160. How was the number of animals determined? Six does not seems to agree with what mentioned in the lines 136 and 137. I have seen similar studies using four mice. Do the authors have any statistical justification?**

Ans: We thank the reviewer for pointing out potential confusion regarding animal numbers and for raising this crucial question concerning sample size determination. To clarify, a sample size of six mice per group (*n* = 6) was utilized across all paw edema assays to balance statistical reliability with the 3Rs ethical principle of reduction. In our preliminary evaluations using the subplantar paw edema model, we observed substantial inter-animal biological variability in baseline and post-challenge paw thickness. Consequently, we determined that using only four mice per group (*n* = 4) rendered our statistical analyses highly vulnerable to individual biological variation and outlying measurements, whereas *n* = 6 consistently provided adequate statistical power to detect dose-dependent and treatment-related changes. Although a formal *a priori* power calculation was not performed, this empirical sample size proved robust and yielded clear, reproducible, and statistically significant results across all crude venom, isolated toxin fraction, and antivenom neutralization experiments, as now explicitly clarified at lines 190 to 194 and lines 264 to 268 (highlighted in blue text) of the revised manuscript.

- **8. lines 178-179. One vial of antivenom should not be appropriate to generalise the conclusions. How do the authors determine if the results were affected by a different lot of antivenoms? I would consider a pool of antivenom more appropriate.**

Ans: We thank the reviewer for this critical methodological consideration regarding the generalizability of our findings. A single vial of freeze-dried hemorrhagic antivenom (FHAV; Lot No. 60-06-0020; Taiwan Centers for Disease Control, Taipei, Taiwan) was intentionally utilized to guarantee strict internal consistency and prevent batch-to-batch noise across all *in vitro* and *in vivo* comparative assays. We fully acknowledge that relying on a single manufacturing lot precludes the evaluation of lot-to-lot variability and limits the immediate extrapolation of our quantitative findings to all commercial FHAV batches. To address this important limitation, we have revised the manuscript text at lines 625 to 627 (highlighted in blue text) to explicitly discuss this caveat, highlighting that future investigations should incorporate multi-lot or pooled-lot antivenom preparations to confirm broader clinical generalizability.

- **9. Figures 1A and 3A are not informative due to quality.**

Ans: We thank the reviewer for the constructive suggestion to enhance the visual clarity of our figures. In response, we have completely removed the low-quality ruler-scaled panels from the original Figures 1A and 3A to prevent confusion and rearranged the figure panels accordingly. Furthermore, all figure panels across the manuscript have been replaced with new, high-resolution vector and bitmap images generated at 1200 dpi to ensure optimal visual legibility and strict compliance with the journal’s publication standards.

- **10. Lines 261–268 and 409–412: FHAV dose is interpreted as the “most critical determinant” of recovery, but this is likely confounding by indication: worse edema receives more vials and also takes longer to resolve. Reframe as association, not causation. Add timing of antivenom, initial severity, progression rate, comorbidities, bite-to-ED time.**

ANS: We thank the reviewer for this constructive comment. In accordance with your recommendation, we have re-included a total of 12 clinical variables, including initial and final Blaylock severity grades, emergency department status, admission duration, surgical history, and baseline comorbidities such as diabetes mellitus and chronic kidney disease, as now presented in the revised Table 1 and at lines 86 to 96 (highlighted in blue text) of the revised manuscript. Using these expanded clinical parameters, we reanalyzed the potential predictors of clinical recovery and found that cumulative FHAV dosage strongly correlates with remission time. Furthermore, we explicitly clarify that the observed correlation between higher cumulative FHAV administration and prolonged remission time represents an observational association rather than direct causation, providing a nuanced interpretation of these statistical results at lines 469 to 490 and lines 642 to 645 (highlighted in blue text) of the revised manuscript.

- **11. Lines 356–380: indirect ELISA only shows binding, not functional neutralization. The manuscript implies “low affinity” explains persistent edema. You need neutralization assays using isolated PLA2, SVSP, and SVMP/CTL ± FHAV, ideally with ED50/effective dose curves.**

Ans: We completely agree with the reviewer that an indirect enzyme-linked immunosorbent assay (ELISA) reflects antigen recognition and relative immunoreactivity rather than functional neutralization or true binding affinity, as now explicitly stated at lines 447 to 451 (highlighted in blue text) of the revised manuscript. Furthermore, we explicitly acknowledge the absence of toxin-specific neutralization assays and median effective dose (ED₅₀) dose-response curves as a study limitation, and we have highlighted these critical evaluations as key directions for future research at lines 587 to 599 (highlighted in blue text) of the revised manuscript.

- **12. Lines 324–332 say FHAV effectively neutralizes edema in mice, but the title/abstract claim edema persists because FHAV poorly targets PLA2. This needs reconciliation. Is the mismatch clinical only? dose-dependent? time-dependent?**
  Ans: We thank the reviewer for highlighting this critical point regarding the apparent discrepancy between preclinical neutralization in mice and persistent edema in patients. We hypothesized that FHAV can neutralize *Protobothrops mucrosquamatus* venom. However, despite its neutralizing capacity, clinical observations indicate that patient swelling does not improve immediately following FHAV administration. Because an antivenom of the same formulation, freeze-dried neurotoxic antivenom (FNAV), fails to neutralize local tissue necrosis (cytotoxicity) induced by *Naja atra* envenomation, questions have arisen regarding whether FHAV exhibits a similar limitation, namely neutralizing most venom components without effectively targeting the specific toxin responsible for local swelling. To address this question, we performed animal experiments and determined that although multiple venom components can induce tissue swelling, PLA₂ plays a major role. Consistent with this finding, FHAV exhibited relatively low immunoreactivity toward PLA₂. *In vivo*, FHAV neutralization of *P. mucrosquamatus* venom demonstrated a clear dose-response relationship. Taken together, these findings suggest that in clinical practice, patients bitten by the Taiwan habu who develop persistent swelling may require continued escalation of antivenom dosing, as now detailed at lines 61 to 64, lines 515 to 517, lines 579 to 581, and lines 640 to 642 (highlighted in blue text) of the revised manuscript.
- **13. Lines 108–114: toxin fractions are “tentatively assigned” by size/retention time, with SVMP and CTL pooled together. For a PLA2-focused paper, this is not enough. Add LC-MS/MS confirmation, SDS-PAGE, enzymatic PLA2 activity, SVMP activity, SVSP activity, and purity estimates. Fraction identity should be confirmed by LC-MS/MS. Retention time alone is insufficient to conclude toxin-family purity.**

Ans: We completely agree with the reviewer that relying solely on estimated molecular mass and chromatographic retention time is insufficient for definitive toxin identification or purity validation. In our study, fraction assignments were based on a previously published, detailed proteomic and chromatographic characterization of *Protobothrops mucrosquamatus* venom established by our research group, in which major toxin families were mapped via LC-MS/MS under identical chromatographic conditions, as reported in Reference 6 and now explicitly detailed at lines 153 to 160 (highlighted in blue text) of the revised manuscript. Furthermore, we explicitly acknowledge this characterization limitation as a study constraint at lines 627 to 631 (highlighted in blue text) of the revised manuscript.

- **14. Lines 193–197 and 501–504: n=3 and only 10 μM is too limited to conclude failure. Test dose-response, venom:inhibitor molar ratios, timing after venom injection, and combination therapy. Otherwise say “not effective under these conditions,” not “failed.**

Ans: We agree with the reviewer that this initial screening phase was not designed as a formal dose-response study and that testing a single concentration is insufficient to conclude intrinsic therapeutic failure. Accordingly, we have thoroughly revised our statements to tone down our conclusions, as now reflected at lines 415 to 420 and lines 558 to 564 (highlighted in blue text) of the revised manuscript.

- **15. Please provide the institutional treatment protocol and criteria used for repeat FHAV administration. Without controlling for indication bias, the conclusion that FHAV dosage predicts remission duration is difficult to interpret.**

Ans: We thank the reviewer for highlighting the need to detail our institutional protocol and address potential indication bias regarding repeat FHAV administration. At our institution, supplemental FHAV dosing is guided by a point-of-care ultrasound protocol that incorporates the Sonographic Assessment of the Rate of Proximal Progression to monitor localized tissue swelling. Clinically, continuous edema expansion reflects an unneutralized venom burden rather than pharmacokinetic failure, which prompts ongoing dose escalation until tissue swelling demonstrably decelerates under serial ultrasonographic evaluation, as noted at lines 528 to 535 (highlighted in blue text) of the revised manuscript. We acknowledge that this clinical algorithm inherently introduces indication bias, as patients with greater initial venom loads and severe local tissue injury naturally receive higher cumulative doses of antivenom and require longer periods to achieve complete tissue repair. Consequently, a higher cumulative FHAV dose serves as an integrated surrogate marker of overall envenomation severity and clinical disease trajectory rather than a direct causal driver of delayed recovery, as clarified at lines 483 to 490 (highlighted in blue text) of the revised manuscript.

- **16. The manuscript does not evaluate delay from envenomation to antivenom administration. This variable should be included in the regression model.**

Ans: We thank the reviewer for this constructive suggestion and clarify that the delay from envenomation to hospital arrival was rigorously evaluated within our clinical cohort study, as detailed in Table 1 and at lines 86 to 96 (highlighted in blue text) of the revised manuscript. Specifically, the time elapsed from bite to emergency department arrival (bite-to-ED time) averaged 3.17 ± 5.47 h (range: 0 to 29 h). Univariate analysis using Pearson’s correlation identified total FHAV dosage (*r* = 0.743, *P* < 0.001), ED stay duration (*r* = 0.544, *P* < 0.001), and final Blaylock level (*r* = 0.375, *P* = 0.004) as factors significantly associated with recovery time; however, the bite-to-ED time was not significantly associated with the duration of edema remission (*r* = -0.029, *P* = 0.422). Furthermore, within a comprehensive multivariable regression model adjusted for 12 clinical variables, the bite-to-ED time was not a significant independent predictor of remission duration (*β* = 0.114, *t* = 1.017, *P* = 0.316). These detailed statistical evaluations and adjusted regression results have been incorporated into the revised manuscript at lines 278 to 305 (highlighted in blue text) to confirm that delayed hospital arrival did not confound the recovery timeline in our cohort.

- **17. Please clarify whether edema resolution was objectively measured or self-reported. Interobserver variability and recall bias should be discussed.**

ANS: We thank the reviewer for highlighting this important methodological detail regarding clinical outcome assessment. In our retrospective cohort, the time to edema resolution was indeed determined through patient self-reports rather than objective clinical measurements, inherently introducing potential recall bias and interobserver variability. As clarified at lines 103 to 107 of the revised manuscript, remission time relied on patient-reported endpoints. Although susceptible to recall bias, this self-reported approach is a pragmatic retrospective surrogate in the absence of standardized objective quantification tools, such as routine serial ultrasonographic or functional tissue measurements, during follow-up. To address this methodological limitation thoroughly, we have expanded the Limitations subsection at lines 609 to 614 (highlighted in blue text) of the revised manuscript to explicitly articulate these confounding factors, including recall bias and interobserver variability, while framing our clinical conclusions within these recognized parameters.

- **18. The authors should correlate edema progression with venom burden whenever possible.**

Ans: We thank the reviewer for this valuable suggestion to explore the correlation between venom burden and edema progression. In our retrospective study of 50 patients envenomed by *Protobothrops mucrosquamatus*, direct real-time serum venom quantification was unavailable; consequently, cumulative FHAV dosage served as an integrated surrogate marker for overall venom burden. Mechanistically, a higher initial venom burden drives rapid, progressive localized swelling, which prompts clinicians to increase FHAV dosing until circulating toxins are neutralized and tissue expansion decelerates. In accordance with your comment, we have thoroughly expanded the Discussion section at lines 483 to 490, and lines 642 to 645 (highlighted in blue text) of the revised manuscript to contextualize cumulative FHAV requirements as an operational measure of total venom burden and to elaborate on the pharmacokinetics and therapeutic dynamics of FHAV in counteracting toxin-mediated edema progression.

- **19. If the PLA₂ fraction contains residual SVSPs or peptides, conclusions become weaker. Please provide purity estimates from SDS‒PAGE and proteomic analysis.**
- **19. Demonstration of catalytic activity would strengthen the conclusion that PLA₂ is the principal edema-driving toxin.**

Ans: We thank the reviewers for these critical points regarding the characterization and functional validation of the isolated PLA₂ fraction. We acknowledge that potential coeluting contaminants from other toxin families, such as SVSPs or small peptides, could influence our results and that demonstrating specific catalytic activity would provide stronger direct evidence that PLA₂ is the primary driver of localized edema. As described in lines 153 to 160 of the revised manuscript, our fraction assignments were based on a previous study (reference 6) in which major snake venom protein families, including PLA₂, SVSP, SVMP, and C-type lectin (CTL), were mapped using LC‒MS/MS under identical chromatographic conditions. However, the specific pooled fractions utilized in the present animal models were not independently reconfirmed by prospective LC‒MS/MS, SDS‒PAGE, or family-specific catalytic activity assays prior to *in vivo* testing. Consequently, we cannot fully exclude the presence of minor coeluting impurities or directly correlate enzymatic activity levels with edema severity. To address this methodological limitation, we have modified our claims throughout the manuscript, characterizing the preparation as a PLA₂-enriched fraction rather than the pure enzyme. Furthermore, as noted in lines 627 to 631 and in the revised Limitations subsection, we have explicitly cited the absence of prospective electrophoretic purity estimates, proteomic reconfirmation, and catalytic activity measurements as a study limitation, while emphasizing the need for future studies to incorporate rigorous purity verification and functional enzymatic neutralization assays.

- **20. Venom and antivenom were mixed before injection. This does not mimic clinical reality, where antivenom is administered after venom dissemination.**

Ans: We thank the reviewer for this important insight into the clinical translation gap in our experimental design. In this study, we utilized a standardized preincubation protocol in which crude venom was mixed with FHAV or SMI prior to injection to determine baseline neutralizing capacity by eliminating confounding pharmacokinetic variables. However, we fully recognize that this preincubation design does not replicate real-world clinical envenomation scenarios, where antivenom is invariably administered after venom dissemination has occurred, as now explicitly discussed at lines 599 to 603 (highlighted in blue text) of the revised manuscript.

- **21. The manuscript says: FHAV neutralizes edema in mice. FHAV inadequately targets PLA₂. Persistent edema occurs clinically. All three cannot be accepted simultaneously without further explanation. The proposed mechanism requires evidence because experimental data demonstrate effective edema neutralization under preincubation conditions**

Ans: We thank the reviewer for identifying this critical logical premise, allowing us to refine our toxicokinetic explanation and explicitly reconcile the apparent discrepancy between preclinical neutralization in mice and persistent edema in patients. Although FHAV effectively neutralizes *P. mucrosquamatus* (Taiwan habu) venom in preclinical models, clinical observations indicate that localized tissue swelling does not resolve immediately following administration, mirroring the persistence of local tissue necrosis during freeze-dried neurotoxic antivenom (FNAV) treatment of *Naja atra* envenomation. To address this mechanism, our animal experiments demonstrated that while multiple venom components induce tissue swelling, PLA₂ plays a primary role, yet FHAV exhibits relatively low immunoreactivity toward PLA₂ compared with SVMPs/CTL. Crucially, effective edema neutralization in mice was achieved using a preincubation protocol wherein FHAV and venom were mixed prior to injection, facilitating optimal binding stoichiometry without temporal delays or tissue diffusion barriers. Conversely, clinical envenomation represents a rescue scenario where PLA₂ rapidly diffuses into subcutaneous tissue to trigger inflammatory cascades prior to intravenous antivenom administration. Given the lower antibody immunoreactivity toward PLA₂ and the high molecular weight of antivenom immunoglobulins such as F(ab')₂ restricting rapid extravasation into swollen interstitial spaces, a kinetic and compartmental mismatch prevents local antibody concentrations from reaching the threshold required to neutralize poorly targeted PLA₂ molecules, thereby allowing localized edema to persist and prompting continued dose escalation in clinical practice. These mechanistic insights and refined toxicokinetic frameworks have been fully integrated into the revised manuscript at lines 61 to 64, lines 515 to 517, lines 579 to 581, lines 599 to 603, and lines 640 to 642 (highlighted in blue text
