# Supplementary material for: Why does local edema persist? Immunological mismatch between phospholipase A2 potency and antivenom affinity in Protobothrops mucrosquamatus envenomation
Source: PLoS Negl Trop Dis. 2026 Sep 3;20(9):e0014689. doi: 10.1371/journal.pntd.0014689 (PMC13541122; doi:10.1371/journal.pntd.0014689)
Supplement: Supplementary file 2 [file pntd.0014689.s003.docx]

Aug 17, 2026

On behalf of all the authors

Editor-in-Chief

PLOS Neglected Tropical Diseases

Dear Editorial Board Members:

Enclosed is a revised version of the manuscript titled **“Why Does Local Edema Persist? Immunological Mismatch between Phospholipase A2 Potency and Antivenom Affinity after *Protobothrops mucrosquamatus* Envenomation**" (Manuscript ID: PNTD-D-26-01063). The comments from the reviewers were valuable and helpful in revising our manuscript, and we appreciate the opportunity for resubmission. We have revised the manuscript on the basis of the recommendations and responded to all the questions raised by the reviewers, including providing point-by-point responses to the comments. Thank you for considering our revised manuscript.

Sincerely,

Cheng-Hsuan Ho, MD, PhD

Attending Physician

Department of Emergency Medicine, Tri-Service General Hospital, National Defense Medical University, Taipei City 114202, Taiwan No. 325, Sec. 2, Cheng-Kung Road, Neihu Dist., Taipei City 11490, Taiwan Tel.: +886-2-87923311-16877 Fax: +886-2-87927034 E-mail: erdoctorho@gmail.com

E-mail: [erdoctorho@gmail.com](mailto:erdoctorho@gmail.com)

# Journal Requirements:

- **Please amend your detailed Financial Disclosure statement. This is published with the article. It must therefore be completed in full sentences and contain the exact wording you wish to be published.i) State the initials, alongside each funding source, of each author to receive each grant. For example: "This work was supported by the National Institutes of Health (####### to AM; ###### to CJ) and the National Science Foundation (###### to AM)."ii) State what role the funders took in the study. If the funders had no role in your study, please state: "The funders had no role in study design, data collection and analysis, decision to publish, or preparation of the manuscript."iii) If any authors received a salary from any of your funders, please state which authors and which funders..If you did not receive any funding for this study, please simply state: u201cThe authors received no specific funding for this work.u201d**

Ans: We thank the editor for these explicit instructions regarding our Financial Disclosure statement, which we have fully updated in accordance with the specified guidelines. The revised Financial Disclosure statement now reads: "This work was supported by research grants from the Tri-Service General Hospital (TSGH-D-113090, TSGH-D-114088, and TSGH-D-115098 to CHH). The funders had no role in study design, data collection and analysis, decision to publish, or preparation of the manuscript. CHH received a salary from the Tri-Service General Hospital in his role as a clinical physician." This complete statement has been incorporated into the revised manuscript at lines 687 to 692.

# Reviewers' Comments: [NOTE: If reviewer comments were submitted as an attachment file, they will be attached to this email and accessible via the submission site. Please log into your account, locate the manuscript record, and check for the action link "View Attachments". If this link does not appear, there are no attachment files.]

Ans: We acknowledge the editor's note and confirm that no attachment files accompanied the decision notification or review comments.

# Figure resubmission:

- **While revising your submission, we strongly recommend that you use PLOS’s NAAS tool (https://ngplosjournals.pagemajik.ai/artanalysis) to test your figure files. NAAS can convert your figure files to the TIFF file type and meet basic requirements (such as print size, resolution), or provide you with a report on issues that do not meet our requirements and that NAAS cannot fix.**

Ans: We thank the editor for this helpful recommendation regarding figure file optimization. We have processed Figures 1 and 2 through the PLOS NAAS tool, and both files successfully passed all technical evaluations, with their dimensions automatically adjusted to satisfy journal requirements. The verified, compliant figure files have been reuploaded to the submission system as Figure 1 revised and Figure 2 revised.
