# Supplementary material for: Why does local edema persist? Immunological mismatch between phospholipase A2 potency and antivenom affinity in Protobothrops mucrosquamatus envenomation
Source: PLoS Negl Trop Dis. 2026 Sep 3;20(9):e0014689. doi: 10.1371/journal.pntd.0014689 (PMC13541122; doi:10.1371/journal.pntd.0014689)
Supplement: Peer review history

# Peer review: Decision Letter 0

**DOI:** 10.1371/journal.pntd.0014689.r001

**Authors:** Wuelton Monteiro

24 Jun 2026

PNTD-D-26-01063

Why Does Local Edema Persist? Immunological Mismatch Between Phospholipase A2 Potency and Antivenom Affinity in Protobothrops mucrosquamatus Envenomation

PLOS Neglected Tropical Diseases

Dear Dr. Ho,

Thank you for submitting your manuscript to PLOS Neglected Tropical Diseases. After careful consideration, we feel that it has merit but does not fully meet PLOS Neglected Tropical Diseases's publication criteria as it currently stands. Therefore, we invite you to submit a revised version of the manuscript that addresses the points raised during the review process.

Please submit your revised manuscript within by Aug 23 2026 11:59PM. If you will need more time than this to complete your revisions, please reply to this message or contact the journal office at plosntds@plos.org. When you're ready to submit your revision, log on to https://www.editorialmanager.com/pntd/ and select the 'Submissions Needing Revision' folder to locate your manuscript file.

Please include the following items when submitting your revised manuscript:

* A letter that responds to each point raised by the editor and reviewer(s). You should upload this letter as a separate file labeled 'Response to Reviewers'. This file does not need to include responses to any formatting updates and technical items listed in the 'Journal Requirements' section below.

* A marked-up copy of your manuscript that highlights changes made to the original version. You should upload this as a separate file labeled 'Revised Manuscript with Track Changes'.

* An unmarked version of your revised paper without tracked changes. You should upload this as a separate file labeled 'Manuscript'.

If you would like to make changes to your financial disclosure, competing interests statement, or data availability statement, please make these updates within the submission form at the time of resubmission. Guidelines for resubmitting your figure files are available below the reviewer comments at the end of this letter.

As the corresponding author, your ORCID iD is verified in the submission system and will appear in the published article. PLOS supports the use of ORCID, and we encourage all coauthors to register for an ORCID iD and use it as well. Please encourage your coauthors to verify their ORCID iD within the submission system before final acceptance, as unverified ORCID iDs will not appear in the published article. Only the individual author can complete the verification step; PLOS staff cannot verify ORCID iDs on behalf of authors.

We look forward to receiving your revised manuscript.

Kind regards,

Wuelton Monteiro, Ph.D.

Section Editor

PLOS Neglected Tropical Diseases

Wuelton Monteiro

Section Editor

PLOS Neglected Tropical Diseases

Shaden Kamhawi

co-Editor-in-Chief

PLOS Neglected Tropical Diseases

orcid.org/0000-0003-4304-636XX

Paul Brindley

co-Editor-in-Chief

PLOS Neglected Tropical Diseases

orcid.org/0000-0003-1765-0002

**Additional Editor Comments (if provided):**

**Journal Requirements:**

If the reviewer comments include a recommendation to cite specific previously published works, please review and evaluate these publications to determine whether they are relevant and should be cited. There is no requirement to cite these works unless the editor has indicated otherwise.

1) Please ensure that the CRediT author contributions listed for every co-author are completed accurately and in full.

At this stage, the following Authors/Authors require contributions: Cheng Hsuan Ho, Yu-Wei Chiang, Yan-Chiao Mao, Chih-Hsiung Hsu, Feng-Chen Chen, Shih-Hung Tsai, and Shing-Hwa Liu. Please ensure that the full contributions of each author are acknowledged in the "Add/Edit/Remove Authors" section of our submission form.

The list of CRediT author contributions may be found here: https://journals.plos.org/plosntds/s/authorship#loc-author-contributions

2) Please upload all main figures as separate Figure files in .tif or .eps format. For more information about how to convert and format your figure files please see our guidelines:

https://journals.plos.org/plosntds/s/figures

3) Please amend your detailed Financial Disclosure statement. This is published with the article. It must therefore be completed in full sentences and contain the exact wording you wish to be published.

i) State the initials, alongside each funding source, of each author to receive each grant. For example: "This work was supported by the National Institutes of Health (####### to AM; ###### to CJ) and the National Science Foundation (###### to AM)."

ii) State what role the funders took in the study. If the funders had no role in your study, please state: "The funders had no role in study design, data collection and analysis, decision to publish, or preparation of the manuscript."

iii) If any authors received a salary from any of your funders, please state which authors and which funders..

If you did not receive any funding for this study, please simply state: u201cThe authors received no specific funding for this work.u201d

4) Thank you for stating "No datasets were generated or analysed during the current study. All relevant data from this study will be made available upon study completion". We strongly recommend all authors decide on a data sharing plan before acceptance, as the process can be lengthy and hold up publication timelines. Please note that, though access restrictions are acceptable now, your entire data will need to be made freely accessible if your manuscript is accepted for publication. This policy applies to all data except where public deposition would breach compliance with the protocol approved by your research ethics board. If you are unable to adhere to our open data policy, please kindly revise your statement to explain your reasoning and we will seek the editor's input on an exemption. Please be assured that, once you have provided your new statement, the assessment of your exemption will not hold up the peer review process.

**Reviewers' Comments:**

Reviewer's Responses to Questions

**Key Review Criteria Required for Acceptance?**

As you describe the new analyses required for acceptance, please consider the following:

**Methods**

-Are the objectives of the study clearly articulated with a clear testable hypothesis stated?

-Is the study design appropriate to address the stated objectives?

-Is the population clearly described and appropriate for the hypothesis being tested?

-Is the sample size sufficient to ensure adequate power to address the hypothesis being tested?

-Were correct statistical analysis used to support conclusions?

-Are there concerns about ethical or regulatory requirements being met?

Reviewer #1: -Are the objectives of the study clearly articulated with a clear testable hypothesis stated?

Yes.

-Is the study design appropriate to address the stated objectives?

Yes.

-Is the population clearly described and appropriate for the hypothesis being tested?

Yes.

-Is the sample size sufficient to ensure adequate power to address the hypothesis being tested?

Yes.

-Were correct statistical analysis used to support conclusions?

Yes.

-Are there concerns about ethical or regulatory requirements being met?

No.

Reviewer #2: Objectives and study design are clearly articulated. Sample sizes and statistics are appropriately described. No ethical or regulatory concerns.

Reviewer #3: Please see comments below.

**********

**Results**

-Does the analysis presented match the analysis plan?

-Are the results clearly and completely presented?

-Are the figures (Tables, Images) of sufficient quality for clarity?

Reviewer #1: In the Results section (lines 330–332), the authors report that while P. mucrosquamatus venom induces potent, dose-dependent swelling, freeze-dried horse antivenom (FHAV) provides effective neutralization of this edematogenic activity. Conversely, the authors pinpoint the limited efficacy of a single dose of varespladib, marimastat, and batimastat in resolving this edema. While this limitation is appropriately addressed in the Discussion, it should be explicitly mentioned in the Results section as well. It has long been established that antivenom dosing must be titrated against the severity of a patient's clinical presentation. Therefore, while these results are original, they are not entirely unexpected. It is highly probable that small-molecule inhibitors would follow the same dose-response principles (i.e., requiring dose escalation corresponding to venom load).

Reviewer #2: Results are clear, images need to be of higher resolution for publication.

Reviewer #3: Please see comments below.

**********

**Conclusions**

-Are the conclusions supported by the data presented?

-Are the limitations of analysis clearly described?

-Do the authors discuss how these data can be helpful to advance our understanding of the topic under study?

-Is public health relevance addressed?

Reviewer #1: The authors highlight the immunological mismatch between toxin potency and antivenom binding specificity as their most significant finding. However, this phenomenon is relatively predictable. The dilutional effect of therapeutic IgG caused by highly abundant, immunodominant (but less toxic) antigens is a well-known hurdle in antivenom therapy. The authors should contextualize this finding more nuances in the text to reflect existing literature on antivenom immunogenicity.

The authors’ conclusion and recommendation that “robust prospective multicenter trials are needed to transition from empirical dosing to standardized, potency-aligned FHAV protocols” is excellent and should be strongly emphasized in the final abstract and conclusion sections.

Reviewer #2: Conclusions are generally supported, but some claims are too bold. Details are provided below (in the summary and comments section).

Limitations are described.

The authors discuss their findings in the context of shaping clinical care.

Reviewer #3: Please see comments below.

**********

**Editorial and Data Presentation Modifications?**

Use this section for editorial suggestions as well as relatively minor modifications of existing data that would enhance clarity. If the only modifications needed are minor and/or editorial, you may wish to recommend “Minor Revision” or “Accept”.

Reviewer #1: 1. Please use standard mathematical and scientific notation throughout the Methods section rather than writing units out in text.

Examples: Use -20°C instead of "minus 20 degrees Celsius"; 0.1% instead of "0.1 percent"; and 20 ± 2 g instead of "20 plus or minus 2 grams".

2. Standardize the notation for critical reagents and manufacturers. Use brackets to denote locations and streamline the text.

Example: Use Gibco (Thermo Fisher Scientific, Waltham, MA, USA) instead of "Gibco at Thermo Fisher Scientific in Waltham, Massachusetts, USA".

3. Remove the duplicated section title found in lines 207–208: "Characterization of Antivenom Binding Profiles via Indirect Enzyme Linked Immunosorbent Assay".

4. Ensure that Latin terms such as in vivo are consistently italicized throughout the entire manuscript.

Reviewer #2: Some panels in a couple of figures are superfluous (details provided below).

Reviewer #3: Please see comments below.

**********

**Summary and General Comments**

Use this section to provide overall comments, discuss strengths/weaknesses of the study, novelty, significance, general execution and scholarship. You may also include additional comments for the author, including concerns about dual publication, research ethics, or publication ethics. If requesting major revision, please articulate the new experiments that are needed.

Reviewer #1: Following the observation that localized swelling progressively worsens despite timely treatment with Protobothrops mucrosquamatus antivenom, the authors suggest that patients may require supplemental dosing to overcome what appears to be weak neutralizing efficacy. The authors test the hypothesis that persistent swelling arises from the synergistic effects of toxins that are not uniformly neutralized by current antivenom therapy. Overall, this manuscript provides highly valuable information for clinicians treating P. mucrosquamatus envenomation. The study is well-conceived, though I have a few questions and comments to help improve the clarity and quality of the manuscript.

Reviewer #2: In this study, the authors aim to understand the underlying causes of persistent edema in patients envenomed with Protobothrops microsquamatus venom despite administration of standard antivenom. They investigate this in a mouse model of edema and fractionate the venom to identify the toxins responsible for this outcome. They note that toxins resulting in local edema are not effectively neutralised by the current antivenom regimen and recommend increasing antivenom doses in parallel with developing antivenoms that better neutralise these toxins (SVSPs and PLA2s). The study is well conducted and logically presented but could benefit from a more extensive setting up of the experimental questions before delving into the data. Some conclusions are also quite strongly presented and require further discussion.

Major comments:

Lines 272- paragraph starts with conclusions but the experimental setup/ hypotheses are not described. Please insert a short paragraph setting up the question and experiment.

Figure 1A – I am uncertain whether figure 1A is needed as the changes at 1-2.5 mm are too small to be visualised by the reader on the ruler, especially at a distance. In addition, the variability in picking the location of the measurements (some at 11 cm, some at 13 or 17 cm) is confusing. I would suggest to just retain figure 1B.

Figure 1B is also fuzzy and of low quality. Please increase the resolution of all figures in the manuscript.

Question: How much venom is there likely to be delivered in a human and how does this scale with the edematogenic mouse model?

Section lines 289-291. As above, this section needs an introductory paragraph stating the hypothesis behind the experiments and describing the experimental setup – for example: ‘To determine the constituents of the venom, we performed reverse phase HPLC…’ etc

Line 297 – section on the mouse paw edema model. A sentence on how the doses and administration of the fractions in the mouse model were decided is needed. How were the doses chosen – based on toxin abundance/ fraction of the total venom in line with previous doses? The rationale behind this is missing. Are the doses for which you see a marked effect in the assay relevant in a patient following envenomation? Please discuss (for example you used 5 ug of PLA2; how much would you normally expect in venom percentage wise and how much would be delivered in a bite?)

Fig 2 – same as above please increase figure resolution

Lines – 317 – Please lead with the question and what is being tested and how – describe venom doses, administration, AV doses, preincubation. Would these doses be relevant in a real life bite scenario?

Line 324 – I recommend changing ‘co-administration’ to ‘the preincubation of venom and antivenom…’ , as co-administration could be interpreted as given at the same time but without a preincubation step.

Fig 3 – as in figure 1, I recommend removing panel A. The images can be included in the supplementary if desired, but panel B is sufficient to make the point presented in A.

SMI paragraph (line 334) – as above, please lead with the hypothesis and experimental setup in a few sentences before describing the results

For the SMI experiments how was the tested 10 uM concentration chosen – were any higher doses used/titrations done?

Line 354 – This statement is too bold – testing at a single concentration may not be enough to support this conclusion. Please rephrase.

Line 358 – Please describe what was done – how was the Elisa set up, what was tested

For the discussion on varespladib – lines 428-430 – please consult these papers regarding inhibition of myotoxins and structural insights:

https://pmc.ncbi.nlm.nih.gov/articles/PMC6868273/

https://pubmed.ncbi.nlm.nih.gov/36403756/

Lines 433-435: The statement is too bold. ‘Paradoxically…. SMIs failed to neutralise’ – at the tested doses, a fact which I believe is important, as no titration experiments with various doses of SMIs have been done. Please modify accordingly. In addition, to make the claim that the venom of P. microsquamatus cannot be neutralised by these inhibitors additional in vitro tests assessing the SVMP and PLA2 activity of the venom in the presence or absence of inhibitors would need to be performed and finally, as noted, a titration of SMIs with higher inhibitor doses in the mouse paw edema model would be required as well.

Line 464 – discussion on supplemental antivenom – how does the venom dose vs antivenom dose translate from the murine model to clinical care? In addition, what is the incidence of adverse reactions in patients for this particular antivenom?

In terms of safety for antivenom administration, when would a second dose of antivenom be recommended? If the MRT is 10 days, is the level of antivenom suboptimal to neutralise remaining toxins?

Lines 485-487 – Please specify how these tests will be done – for example ‘dose escalation studies in preclinical models, synergistic cocktail testing in vitro and in vivo’. While I agree that marimastat and varespladib have proven preclinical efficacy against multiple venoms, a full body of in vitro evidence and in the murine model would be required P. microsquamatus to support clinical translation for these molecules.

Final paragraph on inhibitors in conclusion (lines 518-521) – It cannot be concluded based on a single dose of inhibitor that the inhibitors have failed without testing higher doses. I recommend removing this rationale from the conclusion.

Minor comments:

Line 276 – CV abbreviation was not previously defined

Fig. 1 legend lines 17-19. ‘The results show a clear dose-dependent relationship …. This is a sentence that should not be part of the figure legend, but the results section.

References 20 and 23 are identical

Reviewer #3: This study investigates why local edema often continues to progress after administration of freeze-dried hemorrhagic antivenom (FHAV) in patients bitten by Protobothrops mucrosquamatus (Taiwan habu). Using a combination of retrospective clinical data (50 patients), murine paw edema experiments, venom fractionation, and ELISA immunoprofiling, the authors identified PLA₂ as the most potent edema-inducing toxin family in the venom. However, FHAV showed substantially stronger immunoreactivity toward SVMP/CTL fractions than toward PLA₂ or SVSP fractions, suggesting an immunological mismatch between toxin pathogenicity and antibody recognition. The authors propose that this mismatch may contribute to persistent local swelling despite antivenom treatment and may explain why higher antivenom doses are associated with edema resolution. They further suggest that future antivenom production should include PLA₂ and SVSP as priority immunogens to improve neutralization of local tissue damage.

1. Line 19-24. SVMPs cause more than just disruption of vascular integrity, they also degrade the extracellular matrix of muscle cells leading to poor regeneration. So, their effects must be expanded with appropriate references.

2. Line 81-83. Please provide a reference for support it.

3. Line 81. Please replace minus by –

4. Lines 91-92. Venom enzymes are usually stable, mainly PLA2 which have 7 disulfide bounds. No loss of activity has been reported at room temperature for venom aliquots.

5. Line 141. Please use symbols to replace the words plus and minus

6. Line 141. Why do the authors work with male mice? Based on the findings, the authors showed the female are also an important number of snakebite victims.

7. Lines 159-160. How was the number of animals determined? Six does not seems to agree with what mentioned in the lines 136 and 137. I have seen similar studies using four mice. Do the authors have any statistical justification?

8. lines 178-179. One vial of antivenom should not be appropriate to generalise the conclusions. How do the authors determine if the results were affected by a different lot of antivenoms? I would consider a pool of antivenom more appropriate.

9. Figures 1A and 3A are not informative due to quality.

10. Lines 261–268 and 409–412: FHAV dose is interpreted as the “most critical determinant” of recovery, but this is likely confounding by indication: worse edema receives more vials and also takes longer to resolve. Reframe as association, not causation. Add timing of antivenom, initial severity, progression rate, comorbidities, bite-to-ED time.

10. Lines 356–380: indirect ELISA only shows binding, not functional neutralization. The manuscript implies “low affinity” explains persistent edema. You need neutralization assays using isolated PLA2, SVSP, and SVMP/CTL ± FHAV, ideally with ED50/effective dose curves.

11. Lines 324–332 say FHAV effectively neutralizes edema in mice, but the title/abstract claim edema persists because FHAV poorly targets PLA2. This needs reconciliation. Is the mismatch clinical only? dose-dependent? time-dependent?

12. Lines 108–114: toxin fractions are “tentatively assigned” by size/retention time, with SVMP and CTL pooled together. For a PLA2-focused paper, this is not enough. Add LC-MS/MS confirmation, SDS-PAGE, enzymatic PLA2 activity, SVMP activity, SVSP activity, and purity estimates.

13. Lines 193–197 and 501–504: n=3 and only 10 μM is too limited to conclude failure. Test dose-response, venom:inhibitor molar ratios, timing after venom injection, and combination therapy. Otherwise say “not effective under these conditions,” not “failed.

14. Please provide the institutional treatment protocol and criteria used for repeat FHAV administration. Without controlling for indication bias, the conclusion that FHAV dosage predicts remission duration is difficult to interpret.

15. The manuscript does not evaluate delay from envenomation to antivenom administration. This variable should be included in the regression model.

16. Please clarify whether edema resolution was objectively measured or self-reported. Interobserver variability and recall bias should be discussed.

17. The authors should correlate edema progression with venom burden whenever possible.

18. Fraction identity should be confirmed by LC-MS/MS. Retention time alone is insufficient to conclude toxin-family purity.

19. If the PLA₂ fraction contains residual SVSPs or peptides, conclusions become weaker. Please provide purity estimates from SDS-PAGE and proteomic analysis.

20. Demonstration of catalytic activity would strengthen the conclusion that PLA₂ is the principal edema-driving toxin.

21. Venom and antivenom were mixed before injection. This does not mimic clinical reality, where antivenom is administered after venom dissemination.

22. The manuscript says: FHAV neutralizes edema in mice. FHAV inadequately targets PLA₂. Persistent edema occurs clinically. All three cannot be accepted simultaneously without further explanation. The proposed mechanism requires evidence because experimental data demonstrate effective edema neutralization under preincubation conditions.

**********

PLOS authors have the option to publish the peer review history of their article (what does this mean?). If published, this will include your full peer review and any attached files.

If you choose “no”, your identity will remain anonymous but your review may still be made public.

**Do you want your identity to be public for this peer review?**  For information about this choice, including consent withdrawal, please see our Privacy Policy.

Reviewer #1: **Yes:**  Alison Felipe Alencar Chaves

Reviewer #2: No

Reviewer #3: No

[NOTE: If reviewer comments were submitted as an attachment file, they will be attached to this email and accessible via the submission site. Please log into your account, locate the manuscript record, and check for the action link "View Attachments". If this link does not appear, there are no attachment files.]

**Figure resubmission:**

**Reproducibility:**

To enhance the reproducibility of your results, we recommend that authors of applicable studies deposit laboratory protocols in protocols.io, where a protocol can be assigned its own identifier (DOI) such that it can be cited independently in the future. Additionally, PLOS ONE offers an option to publish peer-reviewed clinical study protocols. Read more information on sharing protocols at https://plos.org/protocols?utm_medium=editorial-email&utm_source=authorletters&utm_campaign=protocols

# Author comment: Author response to Decision Letter 1

**DOI:** 10.1371/journal.pntd.0014689.r002

14 Aug 2026

Attachment Submitted filename: Response_letter_20260811.docx

# Editor report: Decision Letter 1

**DOI:** 10.1371/journal.pntd.0014689.r003

**Authors:** Wuelton Monteiro

17 Aug 2026

PNTD-D-26-01063R1

Why Does Local Edema Persist? Immunological Mismatch Between Phospholipase A2 Potency and Antivenom Affinity in Protobothrops mucrosquamatus Envenomation

PLOS Neglected Tropical Diseases

Dear Dr. Ho,

Thank you for submitting your manuscript to PLOS Neglected Tropical Diseases. After careful consideration, we feel that it has merit but does not fully meet PLOS Neglected Tropical Diseases's publication criteria as it currently stands. Therefore, we invite you to submit a revised version of the manuscript that addresses the points raised during the review process.

Please submit your revised manuscript within by Oct 16 2026 11:59PM. If you will need more time than this to complete your revisions, please reply to this message or contact the journal office at plosntds@plos.org. When you're ready to submit your revision, log on to https://www.editorialmanager.com/pntd/ and select the 'Submissions Needing Revision' folder to locate your manuscript file.

Please include the following items when submitting your revised manuscript:

* A letter that responds to each point raised by the editor and reviewer(s). You should upload this letter as a separate file labeled 'Response to Reviewers'. This file does not need to include responses to any formatting updates and technical items listed in the 'Journal Requirements' section below.

* A marked-up copy of your manuscript that highlights changes made to the original version. You should upload this as a separate file labeled 'Revised Manuscript with Track Changes'.

* An unmarked version of your revised paper without tracked changes. You should upload this as a separate file labeled 'Manuscript'.

If you would like to make changes to your financial disclosure, competing interests statement, or data availability statement, please make these updates within the submission form at the time of resubmission. Guidelines for resubmitting your figure files are available below the reviewer comments at the end of this letter.

As the corresponding author, your ORCID iD is verified in the submission system and will appear in the published article. PLOS supports the use of ORCID, and we encourage all coauthors to register for an ORCID iD and use it as well. Please encourage your coauthors to verify their ORCID iD within the submission system before final acceptance, as unverified ORCID iDs will not appear in the published article. Only the individual author can complete the verification step; PLOS staff cannot verify ORCID iDs on behalf of authors.

We look forward to receiving your revised manuscript.

Kind regards,

Wuelton Monteiro, Ph.D.

Section Editor

PLOS Neglected Tropical Diseases

Wuelton Monteiro

Section Editor

PLOS Neglected Tropical Diseases

Shaden Kamhawi

co-Editor-in-Chief

PLOS Neglected Tropical Diseases

orcid.org/0000-0003-4304-636XX

Paul Brindley

co-Editor-in-Chief

PLOS Neglected Tropical Diseases

orcid.org/0000-0003-1765-0002

**Journal Requirements:**

If the reviewer comments include a recommendation to cite specific previously published works, please review and evaluate these publications to determine whether they are relevant and should be cited. There is no requirement to cite these works unless the editor has indicated otherwise.

1) Please amend your detailed Financial Disclosure statement. This is published with the article. It must therefore be completed in full sentences and contain the exact wording you wish to be published.

i) State the initials, alongside each funding source, of each author to receive each grant. For example: "This work was supported by the National Institutes of Health (####### to AM; ###### to CJ) and the National Science Foundation (###### to AM)."

ii) State what role the funders took in the study. If the funders had no role in your study, please state: "The funders had no role in study design, data collection and analysis, decision to publish, or preparation of the manuscript."

iii) If any authors received a salary from any of your funders, please state which authors and which funders..

If you did not receive any funding for this study, please simply state: u201cThe authors received no specific funding for this work.u201d

**Reviewers' Comments:**

[NOTE: If reviewer comments were submitted as an attachment file, they will be attached to this email and accessible via the submission site. Please log into your account, locate the manuscript record, and check for the action link "View Attachments". If this link does not appear, there are no attachment files.]

**Figure resubmission:**

**Reproducibility:**

To enhance the reproducibility of your results, we recommend that authors of applicable studies deposit laboratory protocols in protocols.io, where a protocol can be assigned its own identifier (DOI) such that it can be cited independently in the future. Additionally, PLOS ONE offers an option to publish peer-reviewed clinical study protocols. Read more information on sharing protocols at https://plos.org/protocols?utm_medium=editorial-email&utm_source=authorletters&utm_campaign=protocols

# Author comment: Author response to Decision Letter 2

**DOI:** 10.1371/journal.pntd.0014689.r004

18 Aug 2026

Attachment Submitted filename: Response_letter_20260817.docx

# Editor report: Decision Letter 2

**DOI:** 10.1371/journal.pntd.0014689.r005

**Authors:** Wuelton Monteiro

19 Aug 2026

Dear Dr. Ho,

We are pleased to inform you that your manuscript 'Why Does Local Edema Persist? Immunological Mismatch Between Phospholipase A2 Potency and Antivenom Affinity in Protobothrops mucrosquamatus Envenomation' has been provisionally accepted for publication in PLOS Neglected Tropical Diseases.

Before your manuscript can be formally accepted you will need to complete some formatting changes, which you will receive in a follow up email. A member of our team will be in touch with a set of requests.

Please note that your manuscript will not be scheduled for publication until you have made the required changes, so a swift response is appreciated.

IMPORTANT: The editorial review process is now complete. PLOS will only permit corrections to spelling, formatting or significant scientific errors from this point onwards. Requests for major changes, or any which affect the scientific understanding of your work, will cause delays to the publication date of your manuscript.

Should you, your institution's press office or the journal office choose to press release your paper, you will automatically be opted out of early publication. We ask that you notify us now if you or your institution is planning to press release the article. All press must be co-ordinated with PLOS.

Thank you again for supporting Open Access publishing; we are looking forward to publishing your work in PLOS Neglected Tropical Diseases.

Best regards,

Wuelton Monteiro, Ph.D.

Section Editor

PLOS Neglected Tropical Diseases

Wuelton Monteiro

Section Editor

PLOS Neglected Tropical Diseases

Shaden Kamhawi

co-Editor-in-Chief

PLOS Neglected Tropical Diseases

orcid.org/0000-0003-4304-636XX

Paul Brindley

co-Editor-in-Chief

PLOS Neglected Tropical Diseases

orcid.org/0000-0003-1765-0002

***********************************************************

# Editor report: Acceptance letter

**DOI:** 10.1371/journal.pntd.0014689.r006

**Authors:** Wuelton Monteiro

Dear Dr. Ho,

We are delighted to inform you that your manuscript, "Why Does Local Edema Persist? Immunological Mismatch Between Phospholipase A2 Potency and Antivenom Affinity in Protobothrops mucrosquamatus Envenomation," has been formally accepted for publication in PLOS Neglected Tropical Diseases.

We have now passed your article onto the PLOS Production Department who will complete the rest of the publication process. All authors will receive a confirmation email upon publication.

The corresponding author will soon be receiving a typeset proof for review, to ensure errors have not been introduced during production. Please review the PDF proof of your manuscript carefully, as this is the last chance to correct any scientific or type-setting errors. Please note that major changes, or those which affect the scientific understanding of the work, will likely cause delays to the publication date of your manuscript. Note: Proofs for Front Matter articles (Editorial, Viewpoint, Symposium, Review, etc...) are generated on a different schedule and may not be made available as quickly.

Soon after your final files are uploaded, the early version of your manuscript will be published online unless you opted out of this process. The date of the early version will be your article's publication date. The final article will be published to the same URL, and all versions of the paper will be accessible to readers.

For Research Articles, you will receive an invoice from PLOS for your publication fee after your manuscript has reached the completed accept phase. If you receive an email requesting payment before acceptance or for any other service, this may be a phishing scheme. Learn how to identify phishing emails and protect your accounts at https://explore.plos.org/phishing.

Thank you again for supporting open-access publishing; we are looking forward to publishing your work in PLOS Neglected Tropical Diseases.

Best regards,

Shaden Kamhawi

co-Editor-in-Chief

PLOS Neglected Tropical Diseases

Paul Brindley

co-Editor-in-Chief

PLOS Neglected Tropical Diseases
